# The mental health impacts of the COVID-19 pandemic among individuals with depressive, anxiety, and stressor-related disorders: A scoping review

**DOI:** 10.1371/journal.pone.0295496

**Published:** 2023-12-14

**Authors:** Christine M. Wickens, Veda Popal, Venesa Fecteau, Courtney Amoroso, Gina Stoduto, Terri Rodak, Lily Y. Li, Amanda Hartford, Samantha Wells, Tara Elton-Marshall, Hayley A. Hamilton, Graham W. Taylor, Kristina L. Kupferschmidt, Branka Agic

**Affiliations:** 1 Institute for Mental Health Policy Research, Centre for Addiction and Mental Health, Toronto, Ontario, Canada; 2 Campbell Family Mental Health Research Institute, Centre for Addiction and Mental Health, Toronto, Ontario, Canada; 3 Dalla Lana School of Public Health, University of Toronto, Toronto, Ontario, Canada; 4 Institute of Health Policy, Management and Evaluation, University of Toronto, Toronto, Ontario, Canada; 5 Department of Pharmacology & Toxicology, University of Toronto, Toronto, Ontario, Canada; 6 Faculty of Liberal Arts & Sciences, Humber College, Toronto, Ontario, Canada; 7 Department of Education, CAMH Library, Centre for Addiction and Mental Health, Toronto, Ontario, Canada; 8 Department of Health Sciences, Lakehead University, Thunder Bay, Ontario, Canada; 9 Department of Psychiatry, University of Toronto, Toronto, Ontario, Canada; 10 Department of Epidemiology and Biostatistics, Schulich School of Medicine and Dentistry, Western University, London, Ontario, Canada; 11 School of Psychology, Deakin University, Geelong, Victoria, Australia; 12 Faculty of Medicine, School of Epidemiology and Public Health, University of Ottawa, Ottawa, Ontario; 13 School of Engineering, University of Guelph, Guelph, Ontario, Canada; 14 Vector Institute for Artificial Intelligence, Toronto, Ontario, Canada; 15 Canada CIFAR AI Chair, Toronto, Ontario, Canada; Universidade Federal do Rio Grande do Sul, BRAZIL

## Abstract

**Objective:**

A scoping review of studies published in the first year of the COVID-19 pandemic focused on individuals with pre-existing symptoms of depression, anxiety, and specified stressor-related disorders, with the objective of mapping the research conducted.

**Eligibility criteria:**

(1) direct study of individuals with pre-existing depressive, anxiety, and/or specified stressor-related (i.e., posttraumatic stress, acute stress) disorders/issues; (2) focus on mental health-related pandemic effects, and; (3) direct study of mental health symptoms related to depression, anxiety, or psychological distress.

**Sources of evidence:**

Database-specific subject headings and natural language keywords were searched in Medline, Embase, APA PsycInfo, and Cumulative Index to Nursing & Allied Health Literature (CINAHL) up to March 3, 2021. Review of potentially relevant studies was conducted by two independent reviewers and proceeded in two stages: (1) title and abstract review, and; (2) full paper review.

**Data charting:**

Study details (i.e., location, design and methodology, sample or population, outcome measures, and key findings) were extracted from included studies by one reviewer and confirmed by the Principal Investigator.

**Results:**

66 relevant articles from 26 countries were identified. Most studies adopted a cross-sectional design and were conducted via online survey. About half relied on general population samples, with the remainder assessing special populations, primarily mental health patients. The most commonly reported pre-existing category of disorders or symptoms was depression, followed closely by anxiety. Most studies included depressive and anxiety symptoms as outcome measures and demonstrated increased vulnerability to mental health symptoms among individuals with a pre-existing mental health issue.

**Conclusion:**

These findings suggest that improved mental health supports are needed during the pandemic and point to future research needs, including reviews of other diagnostic categories and reviews of research published in subsequent years of the pandemic.

## Introduction

In March 2020, the World Health Organization (WHO) declared COVID-19 to be a global pandemic. Public health measures were introduced worldwide in an effort to control spread of the virus. These measures varied from one nation to another and included closure of international borders, shelter-in-place orders, and closure of non-essential businesses, schools and community gathering places. The profound impact of the pandemic and associated public health measures led to a rise in mental health outcomes including elevated symptoms of depression, generalized anxiety, and posttraumatic stress, which was documented by scientists across the globe [1,2].

During the early stages of the pandemic, experts speculated that increased vulnerability to adverse mental health outcomes of the pandemic would be heightened for those with pre-existing mental health issues [3]. Those with pre-existing mental health symptoms or diagnoses were posited to have a heightened sensitivity to stress or depleted coping capacities [4,5], increasing their vulnerability to the impact of pandemic-related stressors. Additionally, people with pre-existing disorders may experience interruptions in treatment or lack access to social supports and/or medication [6,7] or become non-compliant to treatment during the pandemic [6,8].

Exponential growth in research examining the mental health impact of the COVID-19 pandemic prompted the need for a synthesis of current evidence. The current study aimed to conduct a focused scoping review of studies published in the first year of the COVID-19 pandemic that examined vulnerability to mental health outcomes among those with pre-existing mental health issues. To our knowledge, no other reviews of the literature had yet addressed the mental health impacts of the pandemic in this specific population. Recognizing distinct symptomology of each class of psychiatric disorder [9,10], and thus the possibility that individuals with specific mental health symptoms or diagnoses are differentially vulnerable to the effects of the pandemic, the scoping review focused on individuals with pre-existing symptoms of depression, anxiety, and stressor-related disorders. In light of evidence from previous disease outbreaks [11–13], these symptoms were identified early in the pandemic as potential outcomes of concern [3]. The objective of the scoping review was to examine the breadth of research conducted in the pandemic’s first year, including geographical representation, study design, methodology, sample or population of interest, outcome measures and key findings.

## Method

Adoption of a scoping review methodology was selected in light of the unprecedented pace of research publication during the first year of the COVID-19 pandemic, and because of the potentially heterogeneous mix of studies addressing the research question. The review was designed to identify gaps in knowledge, clarify concepts, and summarize evidence to inform policy and practice [14]. The review was guided by the framework originally developed by Arksey and O’Malley [15] and further progressed by the Joanna Briggs Institute [14]. The process involved five stages: (1) identifying the research question and parameters; (2) identifying relevant studies; (3) selecting eligible studies; (4) charting the data, and; (5) collating, summarizing and reporting the results. The Preferred Reporting Items for Systematic reviews and Meta-Analyses extension for Scoping Reviews (PRISMA-ScR) Checklist [16] guided reporting of the findings.

### Identifying the research question

A single research question was identified as the primary focus of the review: How has the COVID-19 pandemic impacted the mental health symptoms of individuals with pre-existing depressive, anxiety, or specified stressor-related (i.e., posttraumatic stress, acute stress) disorders/issues?

### Identifying relevant studies

Articles were identified through searches using database-specific subject headings and keywords in natural language in the following databases: Medline (including Epub ahead of print, in-process and other non-indexed citations), Embase, APA PsycInfo, and Cumulative Index to Nursing & Allied Health Literature (CINAHL).

A medical librarian (TR) developed the search strategies with input from the review team and conducted all searches on October 29, 2020 and again on March 3, 2021. Mental health conditions considered were: pre-existing depressive, anxiety, or specified stressor-related (i.e., posttraumatic stress, acute stress) disorders/issues. To capture articles on pre-existing mental health conditions that may not be described as such, we used natural language search terms such as “improve”, “lessen”, “worsen”, and “exacerbate”, which imply a change over time from a pre-existing state. Due to challenges involved in employing the same terms (i.e., depression and anxiety) in two different concepts within the same search strategy (see inclusion criteria 1 and 3 below), we also designed the strategy to query pre-existing depression, anxiety or stressor-related disorders separately from all other pre-existing mental health conditions. Publication year limits applied were 2019-present, reflecting the onset of the COVID-19 pandemic. No language limits were applied. The full Medline search strategy can be found in Table 1. Records returned from this search were managed in Covidence systematic review software.

**Table 1:** Ovid medline database search strategy: Epub ahead of print, in-process & other non-indexed citations, ovid MEDLINE® daily and ovid MEDLINE® <1946-present>.

1	exp Coronavirus/
2	(coronavir* or corona virus* or nCoV* or COVID or COVID19 or SARS-CoV-2).ti,ab,kf.
3	exp affective disorders/
4	exp anxiety disorders/
5	((preexist* or pre-exist* or exist* or previous* or before or prior or already or chronic or longterm or long-term or lifelong or life-long or history) adj5 (depress* or anxiety or bipolar or dysthymi* or phobia* or panic* or obsessive compulsive or ocd or (mood adj3 disorder*) or (affective adj3 disorder*))).ti,ab,kf.
6	((depress* or anxiety or mood* or affective*) adj3 (diagnos* or disorder*)).ti,ab,kf.
7	(improv* adj3 (mental* or psychiatr* or depress* or anxiety or mood* or affective*)).ti,ab,kf.
8	(worsen* adj3 (mental* or psychiatr* or depress* or anxiety or mood* or affective*)).ti,ab,kf.
9	(relie* adj3 (mental* or psychiatr* or depress* or anxiety or mood* or affective*)).ti,ab,kf.
10	(lessen* adj3 (mental* or psychiatr* or depress* or anxiety or mood* or affective*)).ti,ab,kf.
11	(exacerbat* adj3 (mental* or psychiatr* or depress* or anxiety or mood* or affective*)).ti,ab,kf.
12	mental disorders/ or exp dissociative disorders/ or exp "feeding and eating disorders"/ or exp personality disorders/ or exp "schizophrenia spectrum and other psychotic disorders"/ or exp somatoform disorders/ or exp "trauma and stressor related disorders"/
13	((preexist* or pre-exist* or exist* or previous* or before or prior or already or chronic or longterm or long-term or lifelong or life-long or history) adj5 (personality disorder* or (disorder* adj2 eating) or post-trauma* or posttrauma* or PTSD or complex trauma or psychosis or psychotic or schizo* or manic or mania or mental* or psychiatr*)).ti,ab,kf.
14	((mental* or psychiatr*) adj3 (patient* or inpatient* or outpatient* or facilit* or hospital* or unit* or ward* or setting* or institut*)).ti,ab,kf.
15	((mental* or psychiatr* or obsessive compulsive or ocd or personality disorder* or posttrauma* or posttrauma* or PTSD or complex trauma or (disorder* adj2 eating) or psychosis or psychotic or schizo* or manic or mania) adj3 (diagnos* or disorder*)).ti,ab,kf.
16	(depress* or anxiet* or bipolar or dysthymi* or phobia* or panic* or obsess* or compuls* or OCD or mood* or (affective adj3 disorder*)).ti,ab,kf,hw.
17	or/1-2 [COVID]
18	or/3-11 [preexisting mood or anxiety disorder]
19	or/12-15 [preexisting other MH disorder]
20	17 and 18 [COVID + preexisting mood/anxiety]
21	16 and 17 and 19 [COVID + other preexisting MH disorder + mood/anxiety]
22	20 or 21
23	limit 22 to yr = "2019 -Current”

#### Inclusion criteria

Three criteria were required for inclusion in the review; if any criterion was not met, the study was excluded:

1. Direct study of the population of interest (i.e., primary data from individuals with pre-existing depressive, anxiety, and/or specified stressor-related (i.e., posttraumatic stress, acute stress) disorders/issues);
2. Focus on the mental health effects of the COVID-19 pandemic;
3. Direct study of mental health symptoms related to depression, anxiety, or psychological distress.

#### Exclusion criteria

Articles were considered ineligible if they met either of the following two criteria:

1. Full text was not available in English language.
2. Articles subject to no or minimal peer review (e.g., conference abstracts or proceedings, pre-print articles that have not yet undergone peer review).

### Selecting eligible studies

The review of potentially relevant studies proceeded in two stages: (1) title and abstract review, and; (2) full paper review. To reduce potential bias, the title and abstract of each record were screened by two independent reviewers to identify articles potentially relevant to the scoping review. Conflicts between independent reviewers regarding eligibility of a study for inclusion were discussed and resolved by a minimum of three reviewers, including the Principal Investigator. In determining relevance of articles focused on specific diagnoses, the Diagnostic and Statistical Manual of Mental Disorders (DSM-5) [9] was used; all diagnoses listed under anxiety disorders and depressive disorders were included, as were posttraumatic stress disorder (PTSD) and acute stress disorder.

Based on screening of titles and abstracts, potentially relevant articles were read in full by two independent reviewers. To be included in the final analysis, data related to the mental health symptoms or diagnoses of interest could not be intermixed or merged with data related to other mental health issues that were not relevant to our research question. For example, if the effects of the pandemic were assessed in patients with anxiety, depression or schizophrenia, the article could only be included in the review if analyses examined patients with anxiety and/or depression separately from patients with schizophrenia. A few exceptions were made for studies where only a small minority of the sample was diagnosed with a condition outside of the inclusion criteria; these cases are identified in the data extraction table (see Table 1). Since suicidal ideation is a possible symptom of depression, but is not exclusive to this diagnosis, articles where suicide-related symptoms (e.g., ideation, attempts) were included as a relevant outcome variable were included in the review, while articles in which suicide-related symptoms were listed as the sole pre-existing mental health condition were excluded. In the full text review stage, conflicts between two independent reviewers regarding eligibility of a study for inclusion were resolved by the Principal Investigator. To be included in the final analysis, an article had to be selected in the full paper review stage by at least two reviewers, one of whom had to be the Principal Investigator.

### Charting the data and synthesizing the results

Study details (i.e., location, design and methodology, sample or population, outcome measures, and key findings) were extracted from included studies by one reviewer and confirmed by the Principal Investigator. Data were collated using narrative synthesis.

## Results

**Fig 1:**
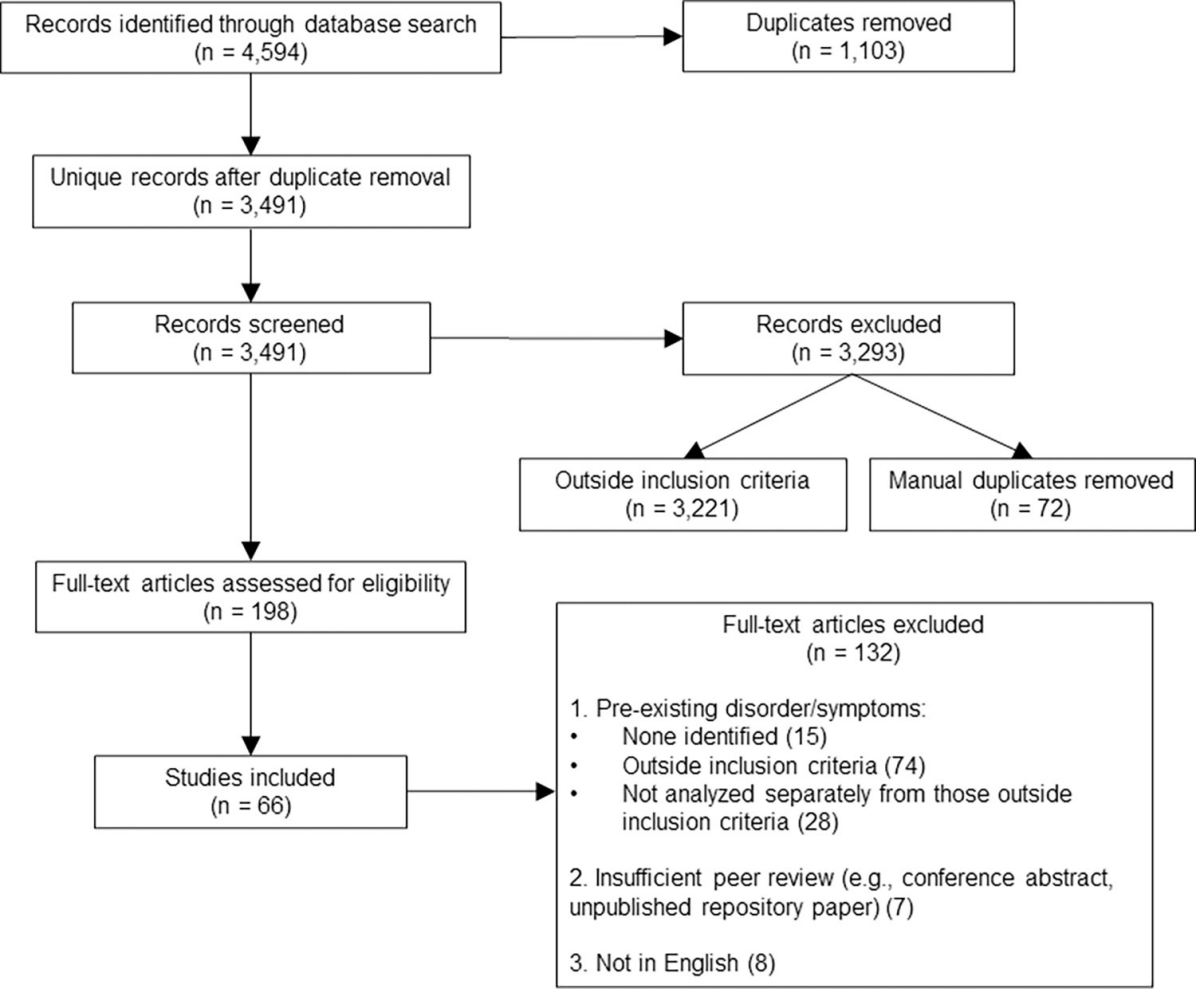
PRISMA flow diagram reporting search results.

The search returned 4,594 records from the four searched databases, resulting in 3,491 records following removal of duplicates. Title and abstract screening identified 198 potentially relevant articles that progressed to full text review. This final stage of review resulted in the identification of 66 articles relevant to the research question (see Table 2). A flow diagram outlining the narrowing of search findings at each stage of the review, including reasons for exclusion at the full text review stage, is provided in Fig 1.

**Table 2:** Data extraction and summary of results (n = 66).

Authors (year)	Location	Design/Method	Aims	Sample	Dependent Measures	Findings
Alonso et al. (2021) [17]	Spain	Cross-sectional online survey	To estimate prevalence of mental disorders in a “sample of healthcare professionals and in subsamples of those with/without prior lifetime mental disorders” and to estimate “associations of individual and professional characteristics, COVID-19 infection status, and COVID-19 exposure with these mental disorders.”	9,138 healthcare workers from 18 healthcare institutions from 6 Autonomous Communities in Spain, of whom 11.2% reported lifetime history of a mood disorder and 35.9% reported lifetime history of an anxiety disorder.	Depression (PHQ-8); Anxiety (GAD-7, number of panic attacks in previous 30 days assessed with item from the WMH-ICS); PTSD (PCL-5)	Chi-square analyses indicate that lifetime history of mood disorder and anxiety disorder are associated with increased prevalence of current symptoms associated with depression, anxiety, panic attacks, and PTSD.
Aragona et al. (2021) [18]	Italy	Pre-post telephone interview (Pre-pandemic measures collected via in-person interview)	“To evaluate perceived mental health during the lockdown period in a sample of immigrants in poor socioeconomic conditions who were in treatment at an outpatient department specifically dedicated to migration and poverty.”	81 outpatients who: “(a) had at least one mental disorder; (b) were immigrants; and (c) were in poor socioeconomic conditions.” Among this sample, multiple mental disorders were reflected including PTSD (34.6% of outpatients), depression (13.6% of outpatients), and anxiety (7.4% of outpatients).	Depression (HDRS item 1); anxiety (HDRS item 10); PTSD (PCL-5, past-week version); COVID-related anxiety (HDRS item 10 modified)	“Mental symptoms were unchanged or improved in many patients, anxiety and nervousness appearing as the most frequently worsened symptoms, but never above 50% (50% and 42%, respectively). Moreover, anxiety for the coronavirus and nightmares were not different in worsened versus stable/improved patients, while of the other symptoms only three (general psychic anxiety, sleep problems, and nervousness) remained significant in the multivariate analysis.” “Worsened patients had higher scores at several psychopathological indexes: post-traumatic thoughts, feelings of irritability, tension and nervousness, sleep problems, depressed mood and anxiety. COVID-19 specific fears were not severe in both groups (it was the variable with the lowest scores) and did not differ significantly.”
Asmundson et al. (2020) [19]	Canada & USA	Cross-sectional online survey	To assess the difference in susceptibility to COVID-19-related stressors across different classes of mental health disorders	Drawn from a quota-based general population sample: 368 with a current primary mood disorder; 700 with a current primary anxiety-related disorder; 500 respondents who did not report a current mental health diagnosis	Depression (PHQ-4); anxiety (PHQ-4); COVID-19-related stress (CSS); stressors associated with self-isolation, and self-isolation distress.	“Respondents with anxiety-related and mood disorders reported significantly higher levels of current anxiety and depression than those with no diagnosis.” “The anxiety-related disorder group reported significantly higher levels of current anxiety and similar levels of current depression compared to the mood disorder group.” “The anxiety-related disorder group reported significantly higher overall COVID-19-related stress…than those in the mood disorder and no mental disorder groups. There were no significant differences between the mood disorder and no mental disorder groups.”
Ballestero et al. (2020) [20]	Brazil	Cross-sectional online survey	“To identify the level of knowledge and readiness of the healthcare providers, as well as to evaluate new preventive practices that have been introduced, psychological concerns, and the impact of the COVID-19 pandemic on pediatric neurosurgical units in Brazil.”	76 active members of the Brazilian Society of Pediatric Neurosurgery	Worsening of previous anxiety symptoms	7.9% reported worsening of previous anxiety symptoms
Barros et al. (2020) [21]	Brazil	Cross-sectional online survey	“To analyze the frequency of sadness, nervousness and sleep pattern changes during the COVID-19 pandemic in Brazil, identifying the most effected [sic] demographic segments.”	Based on a snowballed sample of 45,161 participants, 14.9% of whom reported a history of depression	Depression (During the pandemic, how frequently have you felt sad, crestfallen or depressed?); anxiety (During the pandemic, how frequently have you felt worried, anxious or nervous?)	Feeling sadness/depression all the time or nearly all the time and feeling anxious/nervous all the time occurred more frequently in people with prior diagnosis of depression than among those who did not have prior diagnosis.
Bruffaerts et al. (2021) [22]	Belgium	Cross-sectional online survey	“To investigate the 30-day prevalence of STB [suicidal thoughts and behaviours] and associated risk factors” among clinically active healthcare professionals.	8,758 healthcare professionals, of whom 7.7%, 12.0%, and 2.9% reported a lifetime history of depression, anxiety, and panic attacks, respectively.	Suicidal thoughts and behaviours (modified version of the C-SSRS)	Lifetime emotional problems were strongly associated with suicidal thoughts and behaviours, with the strongest association found for a history of depression.
Bruine de Bruin (2021) [23]	USA	Cross-sectional online survey	To assess “whether older adult age was associated with lower risk perceptions for COVID-19 and with less depression and anxiety.”	6,666 members of the University of Southern California’s (USC) Understanding America Study (UAS). In a pre-pandemic survey, 18% of UAS participants reported a depression diagnosis.	Depression and anxiety (PHQ-4)	A pre-pandemic diagnosis of depression was associated with increased risk of depression and anxiety during the pandemic (PHQ-4 total score, anxiety subscale, depression subscale).
Chaix et al. (2020) [24]	France	Cross-sectional online survey	To assess peritraumatic distress (PD) in at-risk patients in France and identify risk factors for PD and PTSD during the COVID-19 crisis.	1,771 patients in four groups of chatbot users followed for breast cancer, asthma, depression and migraine	Peritraumatic Distress (PDI)	Depression was associated with a significantly higher peritraumatic distress score.
Cheema et al. (2021) [25]	Australia	Cross-sectional online survey	“To quantify stress, anxiety and depression among individuals with Inflammatory bowel disease (IBD) in Australia during the Pandemic.”	352 adults with an underlying diagnosis of IBD living in Australia, 45% of whom reported a pre-existing diagnosis of depression and/or anxiety.	Depression, anxiety, stress (DASS-21 depression, anxiety, and stress subscales); a question regarding perceived impact of pandemic on their diagnosis	More than two thirds of patients with a pre-existing diagnosis of depression and/or anxiety reported worsening of their diagnosis due to the psychological impact of the pandemic. Fig 1 suggests depression, anxiety, and stress higher among IBD patients with pre-existing mental health diagnosis (no statistical analyses reported).
Conte et al. (2020) [26]	Italy	Cross-sectional online survey	To assess emotional and behavioral symptoms of Tourette syndrome among children and adolescents in the early stages of the COVID-19 lockdown and to identify sociodemographic or health-related risk factors.	238 parents of offspring (children, adolescents, or adults) with Tourette syndrome, of whom 51% reported comorbid anxiety symptoms, 16% reported comorbid depression symptoms, and 9% reported comorbid panic symptoms.	Question asking about potential worsening of comorbid symptoms (including anxiety, depression, and panic).	“An increase of anxiety and obsessive-compulsive symptoms (OCS) in 26 and 28% of our sample [was observed].” Worsening of depression and panic was also reported by 14% and 8%, respectively. “However, our findings indicate that anxious-depressive symptoms were outreached by externalizing and behavioral problems.”
Costa et al. (2020) [27]	USA	Cross-sectional online survey	To survey individuals with mental illness about the perceived impact of the pandemic on wellness.	214 members of ForLikeMinds, “an online peer support community dedicated to recovery and wellness of people living with or supporting someone with mental illness, substance use, or a stressful life event” (of which 193 self-identified as living with a mental illness).	COVID-19-related stress (e.g., concern that mental illness would worsen, fear of not getting treatment)	Diagnosis of anxiety was associated with concern that mental illness would worsen, concern that caregivers would be unable to provide support, concern about not getting treatment, concern about getting sick, and coping poorly. Those with an anxiety disorder reported the most concerns. Diagnosis of major depression was associated with fear of running out of medication, fear of getting sick, and feeling less connected to family.
De Pietri & Chiorri (2021) [28]	Italy	Cross-sectional online survey	“To assess the perceived change in anxiety levels and its predictors in a non-clinical, non-infected, home-quarantined Italian sample in the very first weeks of the lockdown.”	593 non-clinical Italian adults who did not report symptoms of COVID-19 at the time of the survey. Reflecting back on how they felt before quarantine, participants completed measures of depression, social anxiety, and health anxiety retrospectively.	Anxiety (BAI)	Bivariate analyses indicated that retrospectively-reported pre-pandemic depression and health anxiety were associated with a perceived increase in anxiety during quarantine. Multivariate analyses indicated that “a perceived increase in anxiety was significantly predicted by higher levels of health anxiety.”
Di Nicola et al. (2020) [4]	Italy	Cross-sectional online survey	“To evaluate the psychological impact of the pandemic in a cohort of remitted patients affected by MDD or BD and to analyze serum 25-hydroxyvitamin D levels” as a predictor of psychological burden.	112 outpatients with DSM-5 diagnosis of MDD or BD regularly referring to the Psychiatric Unit of the Fondazione Policlinico Universitario Agostino Gemelli IRCCS, Universita `Cattolica del Sacro Cuore of Rome.	Global measure of distress, anxiety, depressive symptoms (K10)	A diagnosis of MDD was predictive of more severe psychological distress during the pandemic, compared to BD.
Duarte et al. (2021) [29]	Brazil	Cross-sectional online survey	To evaluate stress perception among Brazilian physical therapists (PTs) during the COVID-19 pandemic and identify psychosocial demands, sociodemographic, professional and clinical factors associated with stress perception.	417 physical therapists, of whom 13.7 percent reported a previous diagnosis of depressive disorder and 23.3 percent reported a previous diagnosis of anxiety disorder.	Perceived stress (PSS)	Previous diagnosis of depressive or anxiety disorder was associated with perceived stress.
Einvik et al. (2021) [30]	Norway	Cross-sectional online or paper survey	“To determine if the prevalence of symptom-defined PTSD 1.5–6 months after confirmed COVID-19 was higher in hospitalized than non-hospitalized subjects” and “to determine risk factors for persistent symptoms of PTSD in COVID-19 survivors.”	125 hospitalized and 458 non-hospitalized COVID-19 survivors.	PTSD symptoms (PCL-5)	Among non-hospitalized COVID-19 survivors, those with a history of depression displayed higher odds of PTSD symptoms.
Fallon et al. (2021) [31]	United Kingdom	Cross-sectional online survey	“To explore the psychosocial experiences of UK women in the early postnatal period (birth to twelve weeks postpartum) during initial government ‘lockdown’ restrictions in the COVID-19 pandemic.”	614 mothers with infants aged between birth and twelve weeks; 11.4% of mothers reported a current clinical diagnosis of depression and 18.4% reported a current clinical diagnosis of anxiety.	Depression (EPDS) and anxiety (STAI-S)	A current clinical diagnosis of depression and of anxiety in step 1 of a hierarchical logistic regression model explained approximately 8% and 10% of the variance in risk of clinically relevant depression, and approximately 7% and 9% of the variance in risk of clinically relevant anxiety. In the fully adjusted models, those without a current diagnosis for depression had reduced odds of both clinically relevant depression and anxiety during the lockdown.
Fountoulakis et al. (2021) [32]	Greece	Cross-sectional online survey	To investigate the prevalence of depression in the adult population aged 18–69 in Greece during the lockdown; the changes in anxiety, distress, suicidal ideation and their association with personal and interpersonal/social variables.	3,399 adults aged 18–69 years, of whom 26.9% reported a history of depression.	Depression (CES-D)	Bivariate analyses indicated that depression and suicidal ideation were more frequently reported by participants with a history of depression compared to those without.
Fu & Zhang (2020) [33]	China	Case study	To demonstrate how “to distinguish between reoccurrent [sic] fever from anxiety/depression induced by the epidemic” and suspected COVID-19 infection.	Male, 18-year-old patient presenting with recurrent sweating, fever, cough and fatigue for 1 month, having a history of being in Wuhan 1 month ago, and a 6-year history of depression.	Depression, anxiety, stress (DASS-21 depression, anxiety, and stress subscales); self- and family-reported depressed mood.	“This case was a patient with depression who was in the maintenance stage of treatment. The depression symptoms reoccurred under the pressure of the COVID-19 epidemic. … The dual effects of depression and stress led to repeated incidence of fever in this case.”
Gao et al. (2020) [34]	China	Cross-sectional online survey	“To investigate the inner beliefs about the COVID-19 pandemic among outpatients with anxiety or depression” and “to identify the factors that influence these inner beliefs.”	“570 outpatients with anxiety or depression; 449 patients’ family caregivers, and; 470 general public” participants	Perceived impact on my pre-existing mental illness; Concern about possible epidemic infection;	43.2% of patients felt their pre-existing mental illness had been impacted by the epidemic. After controlling for age and education, “compared with the general public, patients were less worried about the epidemic but held fewer positive expectations towards COVID-19. The difference between patients and family caregivers showed no statistical significance.”
Ge et al. (2020) [35]	China	Pre-post online survey	To investigate the prevalence “of probable anxiety and probable insomnia and to confirm the risk factors among undergraduate students during the COVID-19 outbreak.”	2,009 undergraduate students from Ocean University who completed self-report screening measures of mental health symptoms (e.g., history of anxiety and depression symptoms, paranoia, social phobia, etc.) upon university entry.	Anxiety (GAD-7)	Based on application of the XGBoost machine learning algorithm, an increase in probable anxiety during the pandemic was associated with a history of anxiety symptoms.
Gobbi et al. (2020) [36]–Study 2 only ^a^	USA	Chart review	“To ascertain factors associated with worsening of psychiatric conditions during the coronavirus disease 2019 (COVID-19) pandemic.”	318 psychiatric patients with “previous diagnosis of major depressive disorder or anxiety disorders (generalized anxiety disorder and PTSD)”.	Worsening of psychiatric symptoms (“clinician report of new symptoms, need to increase or adjust the medication, and referral for a new therapy”).	“Clinicians identified new symptoms in 44% of patients.” Although sleep disturbance was the most common emerging symptom (21% of patients), irritability (7%), panic attacks (5%) and suicidal ideation (<1%), among others, were also reported. “Collectively, clinicians felt the need to make treatment adjustments in almost half of the patients in the form of starting a new medication or treatment modality or adjusting the dose of a currently prescribed medication. …female gender significantly increased the likelihood of a change of medication.”
González-Blanco et al. (2020) [37]	Spain	Matched case-control (secondary subsample analysis of cross-sectional online survey)	“To compare early psychological impact in people with severe mental illness and two control groups (common mental disorders and healthy controls) and to identify risk and protective factors associated with a maladaptive psychological response.”	625 respondents: 125 had severe mental disorders (65 cases of BD, 60 of psychotic disorders; severe mental disorder group–SMD), 250 had other current mental disorders (125 cases of depression, “125 of anxiety (common mental disorder control group–CMD), and 250 had no current or past mental disorders (healthy control group–HC). Subjects in each of the two control groups were matched (ratio 1:2) for sex and age (± 1 year) with the SMD group and, in most cases, also for geographic area.” Data were drawn from a snowballed sample of 21,279 respondents to a cross-sectional online survey.	Depression, anxiety, stress (DASS-21 depression, anxiety, and stress subscales); IES (avoidance and intrusion subscales)	“Bivariate analyses comparing the three groups showed that people with SMD had [significantly higher anxiety, stress, and depression] compared with the HC group, but lower scores compared with the CMD group. People with SMD had lower intrusive thoughts and avoidance scores compared with the CMD group but no differences compared with the HC group.” Regression analyses “showed that COVID-19 was associated with a more intense anxiety response in people with SMD compared with HC. No differences in psychological impact were observed between SMD and CMD groups”
Hamam et al. (2021) [38]	Israel	Cross-sectional online survey	To identify peritraumatic stress symptoms related to COVID-19 among prior trauma survivors, and their association with background characteristics, COVID-19-related stressors, and PTSD symptoms and posttraumatic growth attributed to prior trauma.	528 Israeli adults classified as having been exposed to prior traumatic events based on the Trauma History Screen.	PTSD symptoms (PCL-5)	“More than a quarter of the sample [of trauma survivors] reported having at least one peritraumatic stress symptom related to the pandemic, and 13.4% of the participants had a peritraumatic stress symptom total score of 33 or above, indicating that their symptoms were clinically significant. … Young age and being female were associated with elevated peritraumatic stress symptoms. … being in quarantine and negatively self-rating one’s health status explained elevated levels of peritraumatic stress reactions. … PTSD symptoms resulting from prior trauma were associated with trauma-related symptoms during the COVID-19 pandemic, with effect sizes ranging from medium to large.”
Hamm et al. (2020) [39]	USA	Mixed-methods: interview by telephone and pre-post survey	To assess the experience of “older adults with MDD… during the pandemic, including changes in quantitative depression and anxiety scores from scores obtained before the pandemic.”	73 older adults with MDD recruited from the ongoing Optimizing Outcomes of Treatment-Resistant Depression in Older Adults (OPTIMUM) clinical trial.	Depression (PHQ-9); anxiety (PROMIS)	“Some participants reported they were experiencing increased depression (n = 32) or increased anxiety (n = 33) during the interview. Additionally, 26 reported being isolated. However, overall depression and anxiety scores at the time of the interviews were significantly lower than during the OPTIMUM study baseline and not higher than before the pandemic, indicating that participants [were] not displaying a relapse to pretreatment levels of depression and anxiety during the pandemic. … No increase in suicidal thoughts.” Interviews indicated that “older adults with MDD exhibited resilience in response to the stress of physical distancing. Despite their resilience, most participants’ quality of life was lower, and they worried their mental health would suffer with continued isolation.”
Hao, Tam et al. (2020) [40]	China	Matched case-control survey (COVID-19 patients were also interviewed)	To examine “the neuropsychiatric sequelae of acutely ill patients with coronavirus disease 2019 (COVID-19) infection who received treatment in hospital isolation wards during the COVID-19 pandemic.”	10 acutely ill hospitalized COVID-19 patients with no pre-existing psychiatric illness or unstable medical conditions, 10 age- and gender-matched healthy control participants with no history of psychiatric illness, and 10 age- and gender-matched psychiatric patients previously diagnosed with either MDD–single episode, MDD–recurrent episodes, other anxiety disorders including GAD, panic disorder, or mixed anxiety and depressive disorder based on ICD-10 criteria.	PTSD symptoms (IES-Revised); depression, anxiety, stress (DASS-21 depression, anxiety, and stress subscales); Other psychiatric symptoms (e.g., worried about health, suicidal ideation)	COVID-19 and psychiatric patients were significantly more worried about their health than healthy controls. COVID-19 patients reported higher PTSD symptoms than psychiatric patients and healthy controls, although there was no statistically significant difference between the three groups. COVID-19 and psychiatric patients had higher levels of depression, anxiety and stress than healthy controls, but the difference was not statistically significant.
Hao, Tan et al. (2020) [6]	China	Matched case-control online survey	“To assess and compare the immediate stress and psychological impact experienced by people with and without psychiatric illnesses during the peak of the COVID-19 epidemic with strict lockdown measures.”	76 psychiatric patients from the First People’s Hospital of Chongqing Liang Jiang New Area, China and 109 healthy controls who were age and gender matched. Patients were previously diagnosed with either MDD–single episode, MDD–recurrent episodes, other anxiety disorders including GAD, panic disorder, or mixed anxiety and depressive disorder based on ICD-10 criteria.	PTSD symptoms (IES-Revised); depression, anxiety, stress (DASS-21 depression, anxiety, and stress subscales); Other psychiatric symptoms (e.g., concern about physical health, suicidal ideation)	“Psychiatric patients scored significantly higher on the total IES-Revised, DASS-21 anxiety, depression, and stress subscale. …More than one-quarter of psychiatric patients reported PTSD-like symptoms. …Psychiatric patients were significantly more likely to report worries about their physical health…and suicidal ideation.”
Hodžić et al. (2020) [41]–Case 1 only ^b^	Bosnia & Herzegovina	Case study	To illustrate the psychological impact of the COVID-19 pandemic on at-risk groups of psychiatric patients.	Male, aged 50 years, who is a war veteran with diagnosed complex PTSD.	Suicide attempt	The COVID-19 pandemic resulted in impaired physician-patient communication and led the patient to fear losing his job, which ultimately contributed to suicidal ideation.
Holingue et al. (2020) [42]	USA	Pre-post online survey	“To assess the impact of the COVID-19 pandemic on mental distress in US adults.”	5,325 members of the Understanding America Study (UAS). In pre-pandemic surveys, participants completed the Center for Epidemiologic Studies–Depression Scale (CES-D 8).	Distress (PHQ-4)	Previous symptoms of depression (CES-D 8 scores) were significantly associated with higher mental distress (PHQ-4 scores).
Hölzle et al. (2020) [43]	Germany (country is based on authors’ affiliation)	Cross-sectional (Method unspecified)	“To quantify mental and somatic distress [among psychiatric inpatients two months after lockdown measures] and compare between different diagnostic groups.”	139 psychiatric inpatients of whom, based on ICD-10 criteria, “17 suffered from disorders due to psychotropic substances, 26 were diagnosed with schizophrenia and related disorders, 89 presented with affective disorders, and 7 individuals had other psychiatric diagnoses.”	Stress (PSS); Symptom Severity (Clinical Global Impression)	“There were no significant differences between the dependence, psychosis and affective disorders groups regarding the CGI-score. PSS score [was] highest in the affective disorders group compared to the rest of the sample.” “The subgroup with affective disorders showed the highest correlations between CGI and PSS, whereas no such relationship at all was observed in the schizophrenia and related disorders group.”
Janiri et al. (2020) [44]	Italy	Cross-sectional telephone interview	To identify “risk/protective factors associated with subjective worsening of psychiatric symptomatology during the COVID-19 outbreak in a sample of individuals with PD [Parkinson’s Disease] aged 65 years or older.”	134 individuals with Parkinson’s Disease aged 65 years or older, of whom 75.4% reported lifetime psychiatric symptoms.	Depression and irritability symptoms.	“Up to 22.8% of patients with PD experienced worsening of their psychiatric clinical condition during the COVID-19 outbreak … The most frequent symptom in the group of patients presenting with worsening of psychiatric conditions was depression, which has been reported by up to 82.6% of individuals.”
Jefsen et al. (2020) [45]	Denmark	Chart review	To provide documentation of COVID-19 pandemic-related symptoms of self-harm or suicidality in adults receiving psychiatric treatment in the Central Denmark Region	A keyword search for COVID-19-related symptoms of self-harm/suicidality in clinical notes from in- or out-patients under 18 years of age receiving psychiatric treatment in the Central Denmark Region between February 1 and March 23, 2020 yielded “102 clinical notes from 74 patients.”	Notes indicating symptoms of self-harm/suicidality with a plausible link to the pandemic.	“While suicidal ideation was the most prevalent manifestation, there were also several cases of thoughts of/completed self-harm, suicide attempts, and patients with a passive wish to die of COVID-19. The diagnostic distribution of the patient sample [indicated] that it is the known ‘high risk’ groups for self-harm and suicidality (psychotic disorders, mood disorders, stress-related and adjustment disorders, and personality disorders), which appear to respond to the stress associated with the COVID-19 pandemic with these symptoms/behaviours.”
Jefsen et al. (2020) [46]	Denmark	Chart review	To provide documentation of COVID-19 pandemic-related psychopathology in children and adolescents receiving psychiatric treatment in the Central Denmark Region.	A keyword search for COVID-19-related psychopathology in clinical notes from in- or out-patients under 18 years of age receiving psychiatric treatment in the Central Denmark Region between February 1 and March 23, 2020 yielded “113 clinical notes describing pandemic-related psychopathology in 94 children and adolescents,” including six previously diagnosed with a depressive episode.	Notes indicating psychopathology with a plausible link to the pandemic (e.g., anxiety-related symptoms, self-harm, suicidality)	“Children and adolescents in this investigation that seemingly experienced deterioration tended to fall into two groups with different diagnoses and psychopathological presentations; (a) those with anxiety/stress-related and mood disorders experiencing anxious/nervous symptoms due to fear of the coronavirus, and (b) those with developmental disorders (autism and attention-deficit/hyperactivity disorder) presenting with exacerbated behavioral symptoms due to disruption of habits and familiar structures.”
Karaahmet et al. (2021) [47] ^c^	Turkey	Pre-post in-person interview	“To evaluate whether CAS [the Coronavirus Anxiety Scale] can identify Coronavirus associated anxiety in psychiatric outpatients.”	198 patients who were consecutively admitted to the Psychiatry Department of a State Hospital and had been followed up in a psychiatric outpatient clinic for at least 6 months, who were diagnosed with anxiety disorders and mood disorders before the pandemic.	Change in symptom severity (CGI-I)	Follow-up CGI-I assessments showed that 29.8% of patients had minimally worsened symptoms compared to their pre-pandemic assessment, 21.7% had no change in their symptoms, and 26.8% had improvement at various levels.
Khosravani et al. (2021) [48]	Iran	Cross-sectional survey (online, by telephone, or in-person)	“To validate the Persian-CSS in Iranian patients with ADs [Anxiety Disorders] and OCD… and to compare COVID-19-related stress responses as measured on the CSS among patients with specific ADs and OCD.”	300 individuals with a primary diagnosis of OCD and 310 with a primary diagnosis of an Anxiety Disorder (panic disorder, GAD, special phobia, social anxiety disorder, agoraphobia) recruited from psychiatric hospitals and clinical centers in Iran.	COVID-19 related stress (Persian-CSS)	“Iranian patients with OCD and ADs [Anxiety Disorders] experience distress related to COVID-19 in a similar manner to that shown … in community samples from Canada and the United States.” Patients with GAD and panic disorder “had higher COVID-19-related stress responses than those with SAD [social anxiety disorder] and/or SP [special phobia].”
Kwong et al. (2021) [49]	United Kingdom	Pre-post online survey	“To quantify the prevalence of depression, anxiety and mental well-being before and during the COVID-19 pandemic. Also, to identify groups at risk of depression and/or anxiety during the pandemic.”	“The Avon Longitudinal Study of Parents and Children (ALSPAC) is an ongoing longitudinal population-based study that recruited pregnant women residing in Avon (South-West England) with expected delivery dates between 1 April 1991 and 31 December 1992.” 3,720 individuals in the ALSPAC-parents cohort and 2,973 individuals in the ALSPAC-young cohort participated here. “Generation Scotland: Scottish Family Health Study is a family longitudinal study … [that] recruited across Scotland between 2006 and 2011.” 4,233 individuals from Generation Scotland participated here. The longitudinal studies had measured anxiety and depression symptoms pre-pandemic.	Depressive symptoms (SMFQ in the ALSPAC cohort; PHQ-9 in the Generation Scotland cohort); Anxiety symptoms (GAD-7 in both cohorts)	Pre-pandemic depression and anxiety was associated with greater risk of depression and anxiety during the pandemic.
Lahav (2020) [5]	Israel	Cross-sectional online survey	To explore the contribution of PTSD symptoms as a result of past trauma exposure versus as a result of continuous traumatic stress associated with living in the “Gaza envelope,” in explaining psychological distress (symptoms of peritraumatic stress, anxiety, and depression) in the face of COVID-19.	A convenience sample of 976 Israeli adults, of whom 793 had been exposed to traumatic events. 255 of those exposed to traumatic events had been exposed to rocket attacks (a continuous traumatic event).	Anxiety (BSI-18 anxiety subscale); Depression (BSI-18 depression subscale); Peritraumatic stress symptoms (PCL-5)	“Individuals who had previously been exposed to traumatic events had elevated levels of anxiety, depression, and peritraumatic stress symptoms as related to COVID-19, compared to individuals who had not previously been exposed to traumatic events, even after taking into account demographic characteristics and COVID-19-related stressors. Furthermore, PTSD symptoms subsequent to prior trauma were associated with elevated symptomatology in the face of the pandemic.”
Li et al. (2021) [50]	China	Cross-sectional online survey	“To examine the prevalence of depression and anxiety, and their associations with QOL [quality of life] among clinically stable older patients with psychiatric disorders during the COVID-19 pandemic.”	1,063 clinically stable psychiatric patients aged 50 years and above receiving maintenance treatments in the outpatient departments of four hospitals, of whom 45.6% were diagnosed with MDD, 6.9% with schizophrenia, 5.9% with organic mental disorder, and 41.6% with other disorders.	Depression (PHQ-9); anxiety (GAD-7)	Patients with MDD presented with greater odds of depression, or depression and anxiety combined, than patients with other psychiatric diagnoses.
Liao et al. (2021) [51]	China	Prospective longitudinal online survey (Pre-COVID surveys could be done face-to-face)	“To evaluate the impact of COVID-19 in a subthreshold depressive symptom population” and “explore potential predictors of mental health improvement.”	1,506 individuals aged 18–64 years with subthreshold depressive symptoms (but not lifetime or current MDD) participating in the Depression Cohort in China study, of whom 726 completed the study before the start of the COVID-19 pandemic and 780 began the study before the pandemic but completed during the pandemic.	Depression (PHQ-9); anxiety (GAD-7)	Completing the study intervention during the pandemic, relative to before, was associated with greater odds of probable depression and anxiety at 6-month follow-up. Baseline severity of depression or anxiety showed a strong dose−response gradient with probable depression and anxiety, respectively, at 6-month follow-up. Among those participating during the pandemic, probable depression and anxiety was highest when COVID-19 case counts were highest (between 14 and 27 February, 2020). Individuals reporting greater symptoms of COVID-19-related distress were more likely to have probable depression and anxiety.
Liu et al. (2021) [7]	USA	Cross-sectional online survey	“To determine if health worries due to COVID-19 and grief from experiences of loss because of the COVID-19 pandemic would be associated with higher levels of depression, generalized anxiety, and PTSD symptoms among perinatal U.S. women.”	1,061 U.S. perinatal women over the age of 18 years (starting from the second trimester of pregnancy to those who had given birth in the past six months), of whom “17.5% reported having had a depression diagnosis before pregnancy, 24.5% reported having a diagnosis of generalized anxiety, and 4.1% reported having a diagnosis of PTSD”.	Depression (CES-D); anxiety (GAD-7); PTSD (PCL-C)	“Women with pre-existing mental health diagnoses of depression, anxiety, and PTSD were 1.5 to almost 4 times more likely to endorse current [during pandemic] symptoms above the clinical threshold for symptoms that largely corresponded with these respective diagnoses.”
Mehra et al. (2020) [52]	India	Case study	To present two cases of elderly patients with relapse of depressive disorder who presented to emergency services during the pandemic.	Case 1: 72-year-old male, diagnosed with recurrent depressive disorder 20 years ago. Case 2: 60-year-old female, living alone, diagnosed with recurrent depressive disorder 7 years ago. Both cases maintaining well.	Case 1: extreme anxiety, restlessness, preoccupation with thoughts of COVID-19, developed syndromal depression. Case 2: Worsening depressive symptoms, anxiety about contracting COVID-19, frequent self-care to avoid infection (e.g., washing clothes, bathing, changing sheets frequently)	“Availability of excessive information about COVID-19 in the media, especially about the consequences of the infection for the elderly led to development of initial anxiety. As the anxiety symptoms increased, both patients, who were otherwise maintaining well, developed relapse of symptoms. Other factors which possibly contributed to relapse of symptoms in the second case, was the fact that the person was staying alone. This person was maintaining well, prior to the lockdown; however, [the] lockdown possibly led to marked social isolation, which increased her sense of vulnerability.”
Mink van der Molen et al. (2021) [53]	Netherlands	Pre-post online survey	“To assess the prevalence of anxiety and depression among… breast cancer patients and survivors during the COVID-19 pandemic…[and] the association between… symptoms of anxiety and/or depression and COVID-19-specific concerns, including health care behavior and consumption.”	1,051 (ex-) breast cancer patients enrolled in the ‘Utrecht cohort for Multiple BREast cancer intervention studies and Long-term evaLuAtion’ (UMBRELLA) study, of whom 62.7% experienced symptoms of anxiety and/or depression (as measured by the Hospital Anxiety and Depression Scale subscales) before the COVID-19 pandemic.	Depression and/or Anxiety (Hospital Anxiety and Depression Scale–total score)	“Pre-existent symptoms of anxiety or depression were significantly associated with anxiety and/or depression during COVID-19.”
Mortier et al. (2021) [54]	Spain	Cross-sectional online survey (first phase of planned longitudinal study)	To investigate “the prevalence and correlates of STB [suicidal thoughts and behaviours] among hospital workers during the first wave of the Spain COVID‐19 outbreak.”	5,169 hospital workers recruited from 10 hospitals from four autonomous communities in Spain. Based on the CIDI, 12.1% of respondents reported a lifetime mood disorder prior to the pandemic and 37.9% reported a lifetime anxiety disorder.	Suicidal thoughts and behaviours in the past 30 days, including passive suicidal ideation, active suicidal ideation, suicide plans and/or attempt (C‐SSRS)	Hospital workers with a history of mood disorder and those with a history of anxiety disorder were at increased risk for suicidal thoughts and behaviours during the first wave of the COVID-19 outbreak.
Mortier et al. (2021) [55]	Spain	Cross-sectional telephone survey	“To investigate the prevalence of suicidal thoughts and behaviours… in the Spanish adult general population during the first wave of the… COVID-19 pandemic…and to investigate the individual- and population-level impact of relevant distal and proximal… risk factor domains.”	3,500 non-institutionalised adults recruited through dual-frame random digit dialing of the general population with quotas based on age groups, sex, and autonomous community. Based on the CIDI, 13.6% of respondents reported a lifetime history of depression before the pandemic, 5.5% reported panic attacks, 29.3% reported anxiety, 1.6% reported bipolar disorder, 1.0% reported alcohol use problems, 1.6% reported drug use problems, and 0.8% reported other issues.	Suicidal thoughts and behaviours in the past 30 days, including passive suicidal ideation and active suicidal ideation, plans or attempt (C‐SSRS).	Pre-pandemic depression, anxiety, and panic attacks were all significantly associated with: (1) any suicidal thoughts and behaviour, (2) passive suicidal ideation, and (3) active ideation, plans or attempt. “Associations were generally stronger with active suicidal ideation, plan or attempt than with passive ideation only. … Population-level impact [measured by population attributable risk proportions] was highest for depression and anxiety.”
Padovan-Neto et al. (2023) [56]	Brazil	Cross-sectional online survey	To examine “the psychometric properties of a Brazilian adapted version of the Coronavirus Anxiety Scale (CAS-BR) in a sample of adults in Brazil.”	505 Brazilian adults, of whom 33.1% had previously been diagnosed with an anxiety disorder.	Coronavirus-related fear and anxiety (Coronavirus Anxiety Scale-Brazil)	“Coronavirus anxiety was higher among…participants with a history of anxiety disorder compared to participants without a history of anxiety disorder.”
Pan et al. (2021) [57]	Netherlands	Pre-post online survey (Multiple pre-pandemic data points were averaged to create baseline scores)	To compare “the perceived mental health impact and coping and changes in depressive symptoms, anxiety, worry, and loneliness before and during the COVID-19 pandemic between people with and without lifetime depressive, anxiety, or obsessive-compulsive disorders.”	1,517 respondents “from three cohort studies: the Netherlands Study of Depression and Anxiety (NESDA), Netherlands Study of Depression in Older Persons (NESDO), and Netherlands Obsessive Compulsive Disorder Association Study (NOCDA).” Approximately 78% of participants had been diagnosed with a lifetime mental health disorder including MDD, Dsythymic Disorder, GAD, Panic Disorder, Agoraphobia, Social Anxiety Disorder, and/or OCD.	Depressive symptoms (QIDS), anxiety symptoms (BAI), worry symptoms (PSWQ), perceived mental health impact (9-item scale developed for current study), fear of COVID-19 (6-item scale developed for current study)	Associations of each mental health diagnosis with perceived mental health impact and fear of COVID-19- “were largely similar regarding both direction and magnitude … changes in symptoms from before to during the COVID-19 pandemic were [also] largely similar across disorders except for dysthymic disorder, which showed a relative decrease in depressive symptoms … compared with those in other disorders.” Across disorders, “changes in scores from before to during the pandemic indicated increasing symptom levels in people without mental health disorders, whereas changes of symptom levels were minimal or even negative in individuals with the most severe and chronic mental health disorders. ^d^
Pizzirusso et al. (2021) [58]	USA	Pre-post online survey (Pre-pandemic measures collected via in-person interview)	To examine “psychiatric symptoms in people living with HIV, and their relationship to physical symptomatology and prior psychopathology.”	49 people living with HIV in New York City who were participating in an ongoing longitudinal, observational study conducted by the Manhattan HIV Brain Bank (MHBB). Based on the CIDI, conducted pre-pandemic, 78% of participants had a lifetime history of mood disorder and 61% had a lifetime history of anxiety.	Depression (PHQ-2) and anxiety (GAD-2)	Higher PHQ scores were not associated with prior mood or anxiety disorders. GAD-2 scores were higher with past mood disorders, but not with prior anxiety disorders.
Plunkett et al. (2021) [59]	Ireland	Cross-sectional telephone or in-person interview (patient clinical notes were also reviewed)	“To examine the psychological and social impact of the COVID-19 pandemic on patients with established anxiety disorders during a period of stringent mandated social restrictions.”	30 “patients attending a single sector-based adult community mental health team for the management of an anxiety disorder.” Based on patient clinical notes, 17 patients met criteria for a ‘trigger’ disorder (e.g., OCD), 13 met criteria for a ‘non-trigger’ disorder (e.g., GAD).	Anxiety (BAI, Ham-A); Symptom severity (CGI-S, CGI-I, GAF); Participants’ subjective experience of the impact of the COVID-19 pandemic (e.g., on anxiety and mood symptoms) rated on a 0–10 Likert-type scale.	“Twelve (40.0%) participants described COVID-19 restrictions as having a deleterious impact on their anxiety symptoms. Likert scale measurements noted that the greatest impact of COVID-19 related to social functioning, with a modest deleterious effect on anxiety symptoms noted. Clinician rated data noted that 8 (26.7%) participants had disimproved and 14 (46.7%) participants had improved since their previous clinical review, prior to commencement of COVID-19 restrictions. Conditions associated with no ‘trigger’, such as GAD, demonstrated a non-significant increase in anxiety symptoms compared to conditions with a ‘trigger’, such as OCD.”
Prazeres et al. (2021) [60]	Portugal	Cross-sectional online survey	“To describe the role of spiritual-religious coping regarding fear and anxiety in relation to COVID-19 in healthcare workers in Portugal.”	222 healthcare workers, of whom 32.9% reported a personal history of anxiety.	COVID-19 related fear (FCS) and anxiety (CAS)	Healthcare workers who reported a personal history of anxiety experienced significantly increased levels of COVID-19 related fear and anxiety.
Quittkat et al. (2020) [61]	Germany	Cross-sectional online survey	“To investigate the perceived impact of Covid-19 and its psychological consequences on people with mental disorders.”	2,233 adults, of whom 37.2% reported suffering from a current mental disorder (6.1% GAD, 3.7% panic disorder and agoraphobia, 1.3% illness anxiety disorder, 3.9% social anxiety disorder, 26.2% depression). 26.9% “reported having been affected by a mental disorder in the past.”	Depression (DASS-Depression subscale); Anxiety (PHQ–Panic and Stress Subscale; PSWQ; SIAS; SPS; Whitely Index for assessing attitudes and beliefs of people with illness anxiety)	Compared to retrospectively reported pre-pandemic symptoms, there was a statistically significant increase in symptom severity found for respondents with past or current depression, and a trend toward an increase in symptom severity among those with illness anxiety disorder and GAD. There was a substantial increase in perceived stress levels among respondents with GAD, panic disorder and agoraphobia, and depression, and among healthy controls.
Ravaldi et al. (2020) [62]	Italy	Cross-sectional online survey	“To investigate the psychological impact of the pandemic and ‘lockdown’ on pregnant women.”	737 pregnant women, of whom 32.7% reported a previous diagnosis of anxiety and 9.3% a previous diagnosis of depression.	PTSD (NSESSS); anxiety (STAI-Y)	“Women with self-reported history of anxiety and/or depression were significantly more concerned about COVID-19 and were at a higher risk of developing symptoms of anxiety and posttraumatic stress disorder.”
Rogers et al. (2021) [63]	USA	Pre-post online survey	To examine “(1) adolescents’ perceptions of how their social and emotional lives had changed during COVID-19; and (2) associations between these perceived changes and indices of their mental health, above and beyond their prepandemic mental health status.”	407 adolescents (aged 14–17 years) enrolled in “Project AHEAD (Advancing Health and Education for Adolescent Development), a two-wave longitudinal study of adolescent development”, who had completed measures of depression and anxiety in October 2019.	Depressive symptoms (Children’s Depression Inventory short version); anxiety symptoms (GAD Scale)	Depressive symptoms in October 2019 were the strongest indicator of adolescents’ depressive symptoms in April 2020. Pre-pandemic anxiety symptoms were the strongest indicator of adolescents’ anxiety symptoms 6 months later.
Rutherford et al. (2021) [64]	USA	Pre-post telephone or video-conference interview	To assess “whether exposure to the COVID-19 pandemic in a high infection area worsens mental health among older adults with chronic PTSD”, versus trauma-exposed healthy comparison participants.	76 older adults (50+ years old) participating in an ongoing study of brain aging among individuals with PTSD and trauma-exposed healthy comparison participants (N = 46 PTSD, N = 30 trauma-exposed).	PTSD symptom severity (PCL-5); depressive symptoms (HDRS)	“PCL-5 scores among individuals with PTSD decreased during the COVID-19 pandemic, … whereas the TE [trauma-exposed] group did not change significantly.” “Both the PTSD and TE groups experienced a modest increase in HDRS scores during the pandemic, with the increase among TE subjects being statistically significant.” “Overall no significant differences in HDRS were found between groups, but a race or ethnicity variable was found to moderate HDRS symptom change. Non-black or [non-]Hispanic individuals with PTSD experienced significantly increased HDRS scores during the pandemic compared to black or Hispanic PTSD participants.”
Saraswathi et al. (2020) [65]	India	Pre-post online survey	“To investigate the mental health of undergraduate medical students over a duration of 6 months by analyzing data collected before and during the COVID-19 outbreak in India.”	217 undergraduate medical students in a medical college at Chennai, India. Students self-reported no history of any pre-existing mental health disorders, but mental health status was assessed before the pandemic using the DASS-21.	Depression, anxiety, and stress (DASS-21 depression, anxiety, and stress subscales)	Higher baseline scores of depression, anxiety, and stress were associated with higher levels of the same in follow-up survey.
Serafini et al. (2021) [66] ^e^	USA	Pre-post telephone survey (Both measures collected during pandemic)	“To characterize the psychological impact of the pandemic on Hispanic psychiatric outpatients in East Harlem, while also elucidating socioeconomic variables that might influence their mental health under such stressful conditions.”	Up to 35 patients actively receiving in-person counseling, psychotherapy, and/or psychopharmaceutical treatment from the East Harlem Health Outreach Partnership (EHHOP) Mental Health Clinic who were transitioned to telepsychiatry services. Psychiatric diagnoses within the sample included depressive disorders, anxiety disorders, PTSD, alcohol use disorder, and adjustment disorder. “‘Depressive Disorder’ was the most prevalent disorder across all survey groups, followed by ‘Anxiety Disorder’ and ‘PTSD’”.	Depression (PHQ-2) and anxiety (GAD-2) measured in March then April, 2020. COVID-19-specific depression, anxiety, and worry about infection (items specifically designed for this study) measured once in April or May. Psychological distress (K10+) measured once in May.	“48.57% and 45.71% of participants reported worsened anxiety and depression levels due to the pandemic, respectively. From March to April, PHQ-2 and GAD-2 scores significantly increased.” 57.14% reported being worried about contracting coronavirus. “60% of [K10+] scores reflected serious mental illness. Factors that most influenced K10+ scores included a pre-existing depressive disorder.”
Sharma & Subramanyam (2020) [67]	India	Mixed methods: online survey and telephone interview	To explore whether psychological outcomes “vary across sexual orientation, relationship status, groups with varying risk of COVID-19 complications, history of depression/loneliness, and staying in a state with a high number of COVID-19 cases”.	282 participants completed the online survey which listed the following eligibility criteria: “Indian citizen, presently residing in India, aged 18 years or above, and willing to fill the form in English”. 14 participants completed in-depth interviews, including 4 who had completed the online survey.	Anxiety (GAD-7); depression (CES-D)	Quantitative analyses indicated that a past history of depression or loneliness increased anxiety and depressive symptoms during the lockdown. Qualitative analyses corroborated this finding, with respondents sharing that “stress and anxiety developed during the lockdown had revived old memories of trauma”.
Sherman et al. (2020) [68]	USA	Cross-sectional online survey	To examine the prevalence of mental health difficulties associated with the COVID-19 pandemic, “and risk factors for psychosocial morbidity among community residents in Arkansas.”	591 adults from the ARresearch registry participated. This registry is “comprised of individuals who have expressed potential interest in research participation, and which varies widely with respect to rural vs. urban residence, socioeconomic resources, and racial/ethnic background.”	Depression (PHQ-9); anxiety (GAD-7); trauma-related symptoms (PCL-5)	“Those who had received a prior mental health diagnosis (i.e., mood disorder, anxiety, or PTSD) were at higher risk” for depression, anxiety, and trauma-related symptoms.
Solé et al. (2021) [69]	Spain	Cross-sectional online survey (first phase of planned longitudinal study)	“To evaluate potential differences [in] the effects of the COVID-19 pandemic and lockdown between community controls and patients with a mental illness” and to contrast these effects in individuals diagnosed with depression and/or anxiety versus those diagnosed with bipolar disorder and/or a “schizophrenia-related” disorder. ^f^	619 Spanish citizens including 413 community controls and 206 patients with mental illness drawn primarily from the Bipolar and Depressive Unit of the Hospital Clinic of Barcelona, of whom 14.5% reported an anxiety disorder, 9.7% reported a depressive disorder, 55.3% reported bipolar disorder, and 16.3% reported a schizophrenia-related disorder).	Depressive symptoms (adapted from PHQ-9), anxiety symptoms (adapted from GAD-7), experience of unpleasant events during the lockdown (yes/no), PTSD symptoms (adapted from PTSD Symptom Severity Scale-Revised; asked of those who reported experiencing an unpleasant event), and suicide attempt.	Patients with depression and/or anxiety reported significantly more symptoms of depression and anxiety than patients with bipolar and/or a schizophrenia-related disorder, and suffering more unpleasant events during lockdown. However, there was no difference in the experience of PTSD symptoms or suicide attempts.
Solomou & Constantinidou (2020) [70]	Cyprus	Cross-sectional online survey	To “characterize the psychosocial effects of the COVID-19 pandemic in the general population and to identify risks and protective factors that predict changes in mental health status. In addition, the study investigated compliance with precautionary measures to halt spread of the virus.”	Based on a snowballed sample of 1,642 adults, of whom 276 (16.8%) reported prior anxiety or depression history	Depression (PHQ-9); anxiety (GAD-7)	“Participants with prior psychiatric history are more vulnerable to present severe anxiety and/or depression symptoms compared to the rest of the population.”
Sorokin et al. (2020) [71] ^g^	Russia	Cross-sectional online survey	“To define the structure of anxiety in the population during the epidemic period, as well as to identify the most vulnerable social groups (including individuals with affective disorders) which were most in need of psychological and/or psychiatric help.”	1,957 Russian-speaking respondents aged 18+ years, of whom 29.5% confirmed having been previously diagnosed with an affective disorder (19.8% with major depressive and bipolar affective disorders, 6.0% with anxiety disorders, 3.7% with cyclothymia or dysthymia).	COVID-19-related concerns (developed specifically for this study)	Two COVID-19-related concerns, which were most strongly related to reported psychological stress were observed more frequently in participants who had reported being diagnosed with an affective disorder. Specifically, “‘risk of social isolation’ caused apprehension mostly in individuals with comorbid affective and somatic disorders. At the same time, the ‘lack of medication for daily use’ …was more frequently reported by participants with affective disorders and no comorbidities.” Perception of COVID-19 as a ‘threat to their own life’ was more frequently observed among those reporting an anxiety disorder than a mood disorder.
Sun et al. (2021) [72]	China	Matched case-control (secondary subsample analysis of cross-sectional online survey)	"To investigate the effect of [the] COVID-19 outbreak on stress, anxiety, depression and insomnia in mentally ill patients derived from a national-wide sample in China." ^f^	Drawn from a national sample in China, 244 adults with a mental health diagnosis and 1,116 control participants based on age, gender and region. Among adults with a mental health diagnosis, 1.2% had schizophrenia, 5.3% had bipolar disorder, 35.2% had depression, 40.2% had anxiety, 7.0% had OCD and 11.1% had another diagnosis.	Depression (PHQ-9); anxiety (GAD-7)	Among those with a mental health diagnosis, no to mild levels of anxiety or depressive symptoms in the pre-COVID-19 period (measured retrospectively) were associated with increased risk for worsened anxiety and depressive symptoms during COVID-19 outbreak.
Thombs et al. (2020) [73]	Canada, USA, UK, France	Pre-post online survey	To compare anxiety and depression symptoms among people with systemic sclerosis (an autoimmune disease) and factors associated with changes.	435 Scleroderma Patient-centered Intervention Network (SPIN) Cohort participants were included in the current study. Cohort participants “must be aged ≥18 years and meet 2013 American College of Rheumatology/European League Against Rheumatism criteria for SSc [systemic sclerosis], verified by a SPIN physician”. Cohort participants completed measures of anxiety and depression pre-pandemic.	Change in anxiety (PROMIS—Anxiety); Change in depression (PHQ-8)	Higher pre-COVID anxiety symptoms were associated with smaller increases in anxiety symptoms during the pandemic (see Table 4).
Torales et al. (2020) [74]	Paraguay	Cross-sectional online survey	“To investigate the level of stress perceived by the general population during the current COVID-19 global pandemic and quarantine in Paraguay [and] … factors associated to higher perceived stress.”	2,206 Paraguayan citizens aged 18+ years, of whom 12.4% reported a pre-existing diagnosis of mental disorder including GAD (42.0%), anxiety and depression (23.7%), MDD (17.5%), panic disorder (8.8%), PTSD (0.7%), and others.	Stress (PSS); perceived impact of the pandemic on symptoms	Individuals with a pre-existing psychiatric diagnosis reported higher PSS scores than those without. 63.9% of individuals with a pre-existing psychiatric diagnosis reported their symptoms had worsened, with 42% reporting anxiety.^h^ Among those with a pre-existing psychiatric diagnosis, PSS scores varied as a function of diagnosis, with highest PSS scores reported by those with anxiety and depression.
Vissink et al. (2021) [75]	Netherlands	Cross-sectional online survey	“To explore the effects of the COVID-19 outbreak and measures in patients with pre-existing psychiatric disorders.”	146 patients with a pre-existing psychiatric disorder who had signed broad consent at the Department of Psychiatry of the University Medical Center Utrecht… to be approached for participation in future scientific research.” “The two largest subgroups… were patients with a psychotic disorder (n = 71) and those with an affective disorder (depression or anxiety; n = 86). Smaller subgroups consisted of patients with a developmental disorder (n = 15), a personality disorder (n = 4) and… a variety of other … diagnoses (n = 13)… Comparisons between groups focused on the two largest subgroups.”	Anxiety symptoms (BAI), depressive symptoms (BDI), worry (PSWQ), distress (GHQ), PTSD (PCL-5), effect of the COVID outbreak and measures on the quality of their mental health.	Compared to the psychotic disorder subgroup, patients with affective disorder had significantly higher distress and worry, more severe depressive and PTSD-related symptoms and slightly more severe anxiety symptoms, and reported a greater impact of the pandemic on their mental health.
Werneck et al. (2021) [76]	Brazil	Cross-sectional online survey	"To analyze the associations of physical activity and TV-viewing… patterns during the COVID-19 pandemic quarantine with mental health among Brazilian adults with and without depression."	43,995 adults, of whom 15% reported a previous diagnosis of depression.	Sadness ("During the pandemic period, how often did you feel sad, crestfallen or depressed?"), anxiety ("In the period of the pandemic, how often did you feel worried, anxious or nervous?")	"Physical inactivity and TV viewing were associated with higher odds for negative mental health outcomes, regardless of previous diagnoses of depression." In addition, consistent inactivity was associated with higher sadness during the pandemic, especially among those with previous diagnosis of depression."
Woon et al. (2020) [77]	Malaysia	Cross-sectional online survey	“To accomplish the following: (1) investigate the prevalence and severity of depression, anxiety, and stress in university healthcare workers and (2) determine the association between various factors (demographic, personal, and clinical characteristics; COVID-19-related stressors; and coping), perceived social support, and depression, anxiety, and stress among university healthcare workers in Malaysia after the movement lockdown was lifted. “	399 active full-time staff members at the university hospitals of two public universities in Malaysia, none of whom had a history of pre-existing psychotic disorders, bipolar mood disorder, or illicit drug use or alcohol dependence but 2.8% of whom reported a psychiatrist’s diagnosis of depression or anxiety disorder.	Depression, anxiety, and stress (DASS-21 depression, anxiety, and stress subscales)	Pre-existing depression or anxiety disorder was associated with increased odds of depression, anxiety and stress among healthcare workers, but only the association with stress remained statistically significant after adjusting for sociodemographics, pandemic-related stressors, and perceived social support.
Zhu et al. (2020) [78]	China	Cross-sectional online survey	To investigate the prevalence and factors contributing to anxiety and depression symptoms in first-line anti-epidemic medical staff, and coping styles for these negative emotions.	165 first line medical staff (doctors and nurses) in the designated hospitals and fever clinics of novel coronavirus pneumonia in Gansu Province, of whom 23 (13.9%) reported history of anxiety or depression.	Anxiety and depression (Zung Self-Rating Anxiety/ Depression Scales)	A history of anxiety or depression was a risk factor for anxiety symptoms in doctors during the pandemic, and a risk factor for both anxiety and depression symptoms in nurses.

Abbreviations

BAI = Beck Anxiety Inventory.

BD = Bipolar Disorder.

BSI-18 = Brief Symptom Inventory-18.

CAS = Coronavirus Anxiety Scale.

CES-D = Center for Epidemiologic Studies Depression Scale.

CGI-I = Clinical Global Impression-Improvement.

CGI-S = Clinical Global Impression-Severity.

CIDI = Composite International Diagnostic Interview.

CSS = COVID Stress Scale.

C-SSRS = Columbia Suicide Severity Rating Scale.

DASS-21 = Depression, Anxiety, and Stress Scale.

EDPS = Edinburgh Postnatal Depression Scale.

FCS = Fear of COVID-19 Scale.

GAD = Generalized Anxiety Disorder.

GAF = Global Assessment of Function.

Ham-A = Hamilton Anxiety Rating Scale.

HDRS = Hamilton Depression Rating Scale (also called the Hamilton Rating Scale for Depression).

ICD-10 = 10th revision of the International Statistical Classification of Diseases and related Health Problems.

IES = Impact of Event Scale.

K10 = Kessler 10 Psychological Distress Scale.

MDD = Major Depressive Disorder.

NSESSS = National Stressful Events Survey.

OCD = Obsessive Compulsive Disorder.

PCL = PTSD Checklist.

PDI = Peritraumatic distress inventory.

PHQ = Patient Health Questionnaire.

PROMIS = Patient-Reported Outcomes Measurement Information System.

PSS = Perceived Stress Scale.

PSWQ = Penn State Worry Questionnaire.

QIDS = Quick Inventory of Depressive Symptoms.

SIAS = Social Interaction Anxiety Scale.

SMFQ = Short Mood and Feelings Questionnaire.

SPS = Social Phobia Scale.

STAI = State-Trait Anxiety Inventory.

WMH-ICS = World Mental Health-International College Student.

Notes

Only dependent measures and findings relevant to the current scoping review are included in the table.

^a^ Study 1 was a cross-sectional online survey with 2,734 worldwide psychiatric patients, but diagnoses for these patients were not reported.

^b^ Case 2 describes a patient with a history of bipolar disorder, which falls outside the inclusion criteria of the current review.

^c^ Although the results for bipolar disorder cannot be separated from those of all other diagnoses, these cases represent less than 5% of cases and no significant difference in Coronavirus Anxiety Scale scores by diagnosis was found.

^d^ Results regarding the association of mental health severity and anxiety symptoms (but not depressive or worry symptoms) are based on a sample that includes a small group of individuals with OCD, which falls outside the inclusion criteria for this review.

^e^ Although the results for adjustment disorder and alcohol use disorder cannot be separated from those of all other diagnoses, these cases represent a small portion of the overall sample.

^f^ Results regarding differences between individuals/patients with a mental health diagnosis and those without are excluded from this review because the patient group includes diagnoses irrelevant to the review.

^g^ Although the results for bipolar disorder cannot be separated from those of all other diagnoses, these cases represent a small portion of the overall sample.

^*h*^ Results regarding the association of psychiatric diagnosis with PSS scores are based on a sample that includes a small group of individuals (<8%) with a diagnosis irrelevant to the review.

### Geographical representation

Selected articles were drawn from 26 countries, across five continents (see Table 3). Europe contributed the greatest number of articles (n = 29), with most coming from Italy (n = 6) and Spain (n = 5). This was followed by Asia (n = 16), with most articles coming from China (n = 9), and by North America (n = 15), with most articles coming from the United States (n = 13). These countries were among those with the highest COVID-19 case counts in the first few months of the pandemic [79]. In addition, one article included data from Turkey, one included data from Cyprus, and another included data from Russia, which are countries on the border of Europe and Asia. These numbers have been excluded from the continental counts. Only two studies reported inclusion of data from more than one country. Specifically, Asmundson et al. [19] included data from both Canada and the USA, and Thombs et al. [73] included data from the USA, the UK, Canada, and France.

**Table 3:** Geographical representation of included studies (n = 66).

Continent	n ^a^	%	Country	n ^a^	%
Europe	29 ^b^	43.9	Italy	6	9.0
			Spain	5	7.6
			United Kingdom	3	4.5
			Netherlands	3	4.5
			Germany	2	3.0
			Denmark	2	3.0
			France	2	3.0
			Ireland	1	1.5
			Portugal	1	1.5
			Greece	1	1.5
			Belgium	1	1.5
			Norway	1	1.5
			Bosnia & Herzegovina	1	1.5
Europe & Asia	3	4.5	Türkiye	1	1.5
			Russia	1	1.5
			Cyprus	1	1.5
Asia	16	24.2	China	9	13.6
			India	3	4.5
			Israel	2	3.0
			Iran	1	1.5
			Malaysia	1	1.5
North America	15 ^b^^,^^c^	22.7	United States	13	19.7
			Canada	2	3.0
South America	6	9.0	Brazil	5	7.6
			Paraguay	1	1.5
Australia	4	6.0	Australia	4	6.0

Notes

^a^ Two studies included participants from more than one continent or country. For this reason, sum total n is greater than 66 and sum total percentage exceeds 100%.

^b^ Thombs et al. [73] included participants from Canada, USA, UK, and France, and was added to the continent counts of both North America and Europe and to all four country counts.

^c^ Asmundson et al. [19] included participants from Canada and USA and was added to both country counts.

### Research design

The majority of studies adopted a cross-sectional design (i.e., a single data collection period following onset of COVID-19 cases; n = 42), perhaps because this study design would allow for the most immediate findings. The next most frequently reported study design was a two time-point pre-post design (n = 13). In some cases, these studies were conducted by teams who had relevant pre-pandemic data available (e.g., [18]); in others, participants were asked to report on pre-pandemic mental health retrospectively (e.g., [66]). Other study designs included longitudinal (i.e., more than two data points; n = 1), matched case-control (i.e., two existing groups are compared on the basis of a potential causal attribute; n = 4), chart review (i.e., a review of pre-recorded, patient-centred data; n = 3), and case study (e.g., intensive study of a single patient; n = 3). See Table 4 for a summary of study designs used by relevant studies. Several studies planned to include subsequent data collection phases (e.g., [54,69]). Among the cross-sectional and matched case-control studies (n = 46), the vast majority made some form of comparison between individuals with pre-existing depressive, anxiety, or specified stressor-related disorders or issues and: (a) individuals with no history of mental illness or those who scored below a threshold value on a screening instrument (n = 35) or; (b) those with other mental health diagnoses (n = 10). Among the pre-post and longitudinal studies (n = 14), four examined risk of current psychiatric symptoms among those with versus without history of mental illness, but most studies reported on changes in severity of symptoms (n = 4) or whether the severity of symptoms at time 1 predicted the severity of symptoms at time 2 (n = 6).

**Table 4:** Research design, methodology, sample source, pre-existing disorders/symptoms, and measured outcomes of included studies (n = 66).

**Research Design**	**n**	**%** ^**a**^
Cross-sectional	42	63.6
Pre-post (2 time points)	13	19.7
Longitudinal (>2 time points)	1	1.5
Matched case control	4	6.0
Chart review	3	4.5
Case study	3	4.5
**Methodology** ^**b**^	**n**	**%**
Online survey	50	83.3
Paper-and-pencil surveys	2	3.3
Telephone or videoconference	9	15.0
In-person	3	5.0
**Sample/Population** ^**c**^ **(full sample or control/comparison sample)**	**n**	**%**
General population	26	43.3
Probabilistic or quota-based sampling	5	8.3
Non-probabilistic sampling	21	35.0
Mental health inpatients or outpatients	18	30.0
University students	2	3.3
Healthcare workers	8	13.3
Patients currently or previously diagnosed with COVID-19	2	3.3
Patients with other medical conditions (e.g., breast cancer, Parkinson’s disease, current or recent pregnancy)	10	16.7
**Pre-existing Disorders/Symptoms**	**n**	**%**
Depressive	16	24.2
Anxiety	6	9.1
Depressive and anxiety ^d^	31	47.0
Stressor-related (e.g., post-traumatic stress disorder)	4	6.1
Depressive and stressor-related	1	1.5
Depressive, anxiety, and stressor-related	8	12.1
**Measured Outcomes** ^**e**^	**n**	**%**
Depression	42	63.6
Anxiety	44	66.7
Stress/distress	16	24.2
Post-traumatic stress disorder	14	21.2
Pandemic-specific anxiety	14	21.2
Suicidal thoughts/behaviours	11	16.7
Perceived pandemic impact on mental health	5	7.6
Symptom severity	4	6.1

^a^ Percentages do not sum to 100% due to rounding.

^b^ Excludes chart reviews (n = 3) and case studies (n = 3); therefore, percentage calculated based on n = 60 studies. However, Hölzle et al. [43] did not specify the methodology of their cross-sectional study. Four cross-sectional studies included more than one methodology or allowed participants to choose from one of several methods to access a survey (i.e., online, paper-and-pencil, telephone, and/or in-person interview). Therefore, sum total n is greater than 60 and sum total percentage exceeds 100%.

^c^ Excludes chart reviews (n = 3) and case studies (n = 3); therefore, percentage calculated based on n = 60 studies. However, five studies drew their participants from more than one sample source (e.g., non-probabilistic general sample and mental health inpatients/outpatients). Therefore, sum total n is greater than 60 and sum total percentage exceeds 100%.

^d^ Studies that included either: (a) a measure of depression and a measure of anxiety, or; (b) a single measure of depression/anxiety (e.g., ’affective disorder’ could include both depressive and anxiety disorders).

^e^ Most studies (n = 46) reported more than one measured outcome. Across these studies, 21 different combinations of outcomes were found. Therefore, presented here is the number of studies including the measured outcome, either alone or in combination with other outcomes.

### Methodology

Most studies surveyed research participants, primarily via online survey (n = 50), although several employed alternative approaches; these included data collected through paper-and-pencil surveys (n = 2), over telephone or videoconference (n = 9), and in-person interviews (n = 3). Among cross-sectional surveys, a few studies (n = 3) allowed participants to choose how they accessed the survey during the pandemic (e.g., online, paper-and-pencil, telephone, in-person interview; [30,48,59]). One cross-sectional study used “a convergent mixed-methods approach” ([67], p. 4) including both in-depth qualitative telephone interviewing and online survey. Hölzle et al. [43] conducted a cross-sectional survey but did not specify the method by which data were collected. See Table 4 for a summary of methodologies used by relevant studies.

### Sample/Population

Participants were drawn from a diversity of sources. Twenty-six studies were conducted with samples (or control/comparison samples) clearly drawn from the general population. Of these, five studies used probabilistic samples or quota-based sampling (e.g., [19,23]), four of which recruited participants from existing panels or ongoing studies and one recruited through random digit dialling. Of the studies drawn from the general population that were non-probabilistic (e.g., [56,61]), most used social media, online or conventional media advertising or snowballing techniques to recruit participants; however, some recruited all or a portion of their participants from existing panels or ongoing studies. Eighteen studies were conducted with samples of mental health inpatients or outpatients recruited from hospitals, clinics, mental health programs or ongoing studies. Other special samples, either as a primary or comparison group, included university students (n = 2; [35,65]), healthcare workers (n = 8; e.g., [77,78]), patients currently or previously diagnosed with COVID-19 (n = 2; [30,40]), and patients with other medical conditions such as breast cancer, Parkinson’s disease, and current or recent pregnancy (n = 10; e.g., [7,44,53]). These special samples were typically recruited from existing institutions (e.g., hospitals, universities), although advertising and snowballing techniques were used to recruit half of the studies of patients with other medical conditions. Table 4 summarizes the various sources from which relevant studies drew their study samples.

### Pre-existing disorders/symptoms

Among the included studies, the most commonly reported pre-existing category of disorders or symptoms was depressive disorders (n = 51; e.g., major depressive disorder), followed closely by anxiety disorders (e.g., n = 40; generalized anxiety disorder, panic disorder). Seven studies reported a pre-existing condition that could be labelled as depression *or* anxiety (e.g., affective disorder). A total of 62 studies included participants with a history of or pre-pandemic experience of depression and/or anxiety or related symptoms. Far fewer studies examined the stressor-related disorders of PTSD or acute stress disorder or related symptoms (n = 13), and most of these studies (n = 9) also included participants with a history of or pre-existing depressive and/or anxiety symptoms. See Table 4 for a summary of pre-existing disorders or symptoms examined by relevant studies. Most studies (n = 48) focused on individuals with a diagnosed disorder, although in about half of these studies the diagnoses were reported by the participant themselves (n = 21). For this reason, diagnoses provided in studies of patient populations, chart reviews, or case studies were likely more reliable. Other studies (n = 19) relied on self-reporting of symptoms or screening measures to identify individuals with a history of mental health disorder or pre-existing symptoms. Aragona et al. [18] included symptom measures in a sample of diagnosed patients.

### Outcome measures

Most studies (n = 46) reported more than one measured outcome. Across these studies, 21 different combinations of outcomes were found, with no more than four studies sharing the same combination of outcomes. The only exception was the most frequently reported combination of depression and anxiety. Two studies included an outcome measure of depression alone and three included a measure of anxiety alone. Forty studies included measures of both depression and anxiety, with 22 of these studies including additional outcomes as well. Depression and anxiety were assessed using a broad diversity of predominantly well-validated and reliable measures (e.g., Brief Symptom Inventory (BSI-18); Depression, Anxiety, and Stress Scale (DASS-21); Generalized Anxiety Disorder (GAD-7) Scale; one of several versions of the Patient Health Questionnaire (PHQ)). In three of these studies, analyses treated anxiety and depression as a single construct [23,42,53]. Either alone or in combination with other outcome measures, about one quarter of studies included measures of general stress or distress (n = 16) and another quarter of studies included measures of PTSD symptoms (n = 14); only four studies included both types of stress-related measures [6,37,40,75]. General stress or distress was most frequently measured by the DASS-21 stress subscale (n = 7) or the Perceived Stress Scale (n = 3), and PTSD symptoms were most frequently measured by the PTSD Checklist (PCL-5 or PCL-C; n = 8) or the Impact of Event Scale (IES; n = 3). Either alone or in combination with other outcomes, about one quarter of studies also measured anxiety specific to the pandemic (n = 14), relying on items developed specifically for their survey or interview or on newly developed screening scales such as the COVID Stress Scale (e.g., [19,48]) or Coronavirus Anxiety Scale (e.g., [56,60]). Eleven studies examined suicidal thoughts and behaviours, commonly drawing on patient files [41,45,46] or responses to the Columbia Suicide Severity Rating Scale [22,54,55]. Five studies developed items asking participants to rate perceived impact of the pandemic on their mental health (e.g., [34,57]) and four studies included a measure of symptom severity (i.e., Clinical Global Impression–impression and severity subscales; e.g., [47,59]). Table 4 includes a summary of measured outcomes reported by relevant studies.

### Key findings of eligible studies

In reviewing study findings, it was clear that the vast majority of studies found an association between a pre-existing mental health issue and increased vulnerability to adverse mental health outcomes during the COVID-19 pandemic. Depending on study design, these studies noted either: an increase in symptoms during the pandemic among those with pre-existing symptoms; an increased risk of developing symptoms among those with a history of the condition or pre-existing symptoms, or; a higher prevalence or severity of symptoms among those with a history of the condition or pre-existing symptoms compared to either those without such a background or those with an alternative mental or physical health condition.

Among individuals with a history of or pre-existing depression or depressive symptoms, the greatest number of studies detected an increase in or greater risk of depression (n = 20), followed closely by an increase in or greater risk of anxiety (n = 13). Pre-existing depression or depressive symptoms were also associated with increased or greater risk of symptoms of stress (n = 5), COVID-related stress/anxiety (n = 3), PTSD (n = 2), suicidal thoughts or behaviour (n = 5), and a perception that the pandemic had impacted one’s mental health (n = 1). Results were similar among individuals with a history of or pre-existing anxiety or anxiety symptoms, for whom the greatest number of studies identified increased or greater risk of anxiety (n = 13), followed by an increase in pandemic-specific stress/anxiety (n = 6). Associations with increased symptoms of depression (n = 4), suicidal thoughts or behaviour (n = 3), stress (n = 1), and PTSD (n = 1) were also found. Additional studies of pre-existing depression or depressive symptoms (n = 5) and of pre-existing anxiety or anxiety symptoms (n = 1) identified a positive association with during-pandemic symptoms of depression, anxiety, *or* stress (treated as a single outcome). Analyses in several studies considered individuals with pre-existing depression *or* anxiety (treated as a singular condition), or depressive *or* anxiety symptoms. Such studies identified an association with depressive (n = 9), anxiety (n = 9), and stress (n = 8) symptoms, as well as PTSD symptoms (n = 4), pandemic-related stress/anxiety (n = 4), perceived negative impact on one’s mental health (n = 3), depressive *or* anxiety symptoms (treated as a single outcome; n = 1), and suicidal thoughts or behaviour (n = 1). Analyses in two additional studies (i.e., [66,68]) treated individuals with pre-existing depression *or* anxiety *or* PTSD (treated as a singular condition) and found associations with increased risk of depressive, anxiety, and PTSD symptoms during the pandemic.

A few studies provided a comparison of during-pandemic symptoms among individuals with a history of or pre-existing depression or depressive symptoms and those with a history of or pre-existing anxiety or anxiety symptoms. Asmundson et al. [19] reported that a group with anxiety-related disorders had higher levels of anxiety and COVID-19 related stress/anxiety than a mood-disorder group, but similar levels of depression. Costa et al. [27] found that within an online peer support community of individuals living with mental illness, those with an anxiety disorder reported the most stresses or concerns about the COVID-19 pandemic. Sorokin et al. [71] found that perceiving COVID-19 as a threat to one’s own life was more common among those reporting an anxiety disorder than a mood disorder. However, based on data from ongoing cohort studies, Pan et al. [57] found that perceived mental health impact, fear of COVID-19, and changes in symptoms from before to during the pandemic were largely similar across various mood and anxiety disorders.

Aside from studies focusing on pre-existing depression or anxiety, several studies found that a history of pre-existing trauma or PTSD, or related symptoms, was associated with increased risk of depressive (n = 2), anxiety (n = 1), and PTSD (n = 3) symptoms and suicidal thoughts or behaviour (n = 2) during the pandemic. Also, Saraswathi et al. [65] reported that higher pre-pandemic stress scores were associated with higher levels of stress during the pandemic.

Findings both within and across studies were not entirely consistent; a small number of studies (n = 13) reported no difference in vulnerability for at least one tested symptom among those with pre-existing depression, anxiety, or stressor-related disorders or symptoms. However, all but two of these studies simultaneously found evidence of increased vulnerability to other symptoms (e.g., [7,28,35]). Only five studies found any evidence of a *reduced* vulnerability for mental health symptoms among individuals with pre-existing mental health issues, or among individuals with more severe versus less severe pre-existing symptoms (i.e., [34,57,64,72,73]). However, as with the few cases of null effects, findings of reduced vulnerability were typically found for select symptoms only and accompanied demonstrated effects of increased vulnerability (e.g., [34,57]). Sun et al. [72] is an exception; among those with a mental health diagnosis, no to mild levels of anxiety or depressive symptoms before the pandemic (measured retrospectively) were associated with increased risk for worsened anxiety and depressive symptoms during the pandemic. Of the studies that identified a reduced vulnerability for mental health symptoms among those with a pre-existing mental health issue or more severe symptoms, one adopted a cross-sectional design, one adopted a matched case-control design, and three were pre-post studies.

## Discussion

This scoping review of literature published in the first year of the COVID-19 pandemic assessed its impact on symptoms related to depression, anxiety, and psychological distress among individuals with pre-existing depressive, anxiety, or specified stressor-related (i.e., posttraumatic stress, acute stress) disorders/symptoms. The review identified 66 relevant articles with data from 26 countries, although countries with higher case counts in the earliest months of the pandemic appeared to publish a greater number of relevant studies. The majority of published studies adopted a cross-sectional design. About one quarter of studies adopted a two time-point pre-post design, either relying on available pre-pandemic data or asking study participants to report on pre-pandemic mental health retrospectively. The current review, therefore, underscores a need for longitudinal designs with well-characterised sampling frames that can more accurately monitor changes in mental health symptoms over time using validated measures. The small number of pre-post studies relative to cross-sectional studies precludes robust conclusions concerning the role of study design in research findings; however, it should be noted that while just one of 42 cross-sectional studies found possible evidence of reduced vulnerability to mental health symptoms among those with a pre-existing mental health issue or more severe symptoms, three of just 13 pre-post studies did so, highlighting further the need for longitudinal study of this research question. An overwhelming majority of studies were conducted via online survey, which was likely the most convenient and potentially the only viable option for many studies given physical distancing requirements. Depending on availability of Internet access, reliance on online surveys may have introduced selection bias into the results, perhaps in some jurisdictions more than others. Almost half of studies drew their sample, or a control sample, from the general population, with only a small portion of these using probabilistic samples or quota-based sampling. The remainder of studies focused on special populations, primarily mental health inpatients or outpatients recruited from hospitals, clinics, mental health programs or ongoing studies. The most commonly reported pre-existing category of disorders or symptoms was depressive disorders, followed closely by anxiety disorders. Likewise, the majority of studies included depressive and anxiety symptoms as outcome measures. About one quarter of studies assessed general stress or distress and another quarter assessed PTSD symptoms. Most studies identified an association between a pre-existing mental health issue, predominantly depression and/or anxiety, and increased vulnerability to adverse mental health symptoms during the COVID-19 pandemic.

### Strengths and limitations

These results are generally consistent with other reviews examining this issue [80–82]. However, the current review assessed the first full year of the COVID-19 pandemic as opposed to the first several months, which is a limitation of the early reviews (e.g., [81]). The current review also focused on depressive, anxiety, and specified stressor-related disorders, all examples of ‘common mental disorder’. This is important because different disorders are associated with different symptoms and treatment needs, which may be differentially impacted by the pandemic and associated public health restrictions. There is emerging evidence to suggest that the mental health impact of the pandemic differed for individuals with severe mental disorder (e.g., a psychotic disorder) compared to those with a common mental disorder [37]. Depressive, anxiety, and specified stressor-related disorders were selected as the focus of the current review because literature from previous disease outbreaks and early literature of the general population at the onset of the COVID-19 pandemic identified an increase in symptoms typical of these disorders [11–13].

Limitations of the review included its focus on DSM-5 classifications [9] to define depressive and anxiety disorders. ICD-10 classifications [10], which are commonly used by mental health professionals outside of North America, do not align perfectly with the DSM-5. Thus, the review would likely have included additional articles had the DSM-5 not been used to interpret inclusion criteria; however, it cannot be determined if the results of the review would have differed substantially. Although the current review focused on specific mental health issues, it was not restricted to mental health patients only; it included studies of individuals with a history of or pre-existing mental health disorder or self-reported symptoms associated with these disorders, allowing assessment among individuals without a formal or current diagnosis. There was no requirement that studies adopt diagnostic instruments rather than rely on brief mental health screening scales, which are intentionally sensitive, resulting in higher risk of false-positive ratings, overestimation of prevalence, and lack of concordance with functional impairment [83]. Likewise, where diagnoses were considered, there was no requirement that diagnoses be confirmed by clinicians or chart review rather than be self-reported by patients. Given the limited number of studies that included diagnostic instruments or clinician-confirmed diagnoses, it was important to use broad inclusion criteria in order to map relevant literature that was available in the first year of the pandemic. A related limitation that is characteristic of scoping review methodology is the absence of quality assessment, which would specify the level of methodological rigour associated with each study. While scoping reviews provide a map or an overview of the evidence, they do not aim to produce a synthesized answer to a particular question. For this reason, assessment of methodological limitations or risk of bias in the evidence is not conducted as part of a scoping review [84,85].

### Implications

Research findings summarized by the review have important implications. Tracking the worsening of symptoms during the COVID-19 pandemic among individuals with a pre-existing mental health issue can be used to promote action in improving the mental healthcare system, better equipping it to address concerns from its existing patient population and mitigate further mental deterioration [36]. In Canada, access to publicly funded mental healthcare was limited prior to the pandemic, which only exacerbated the situation. Mental health research funding was not proportionate to the burden of mental illness on the population [86]. The rise in depressive, anxiety, and stressor-related symptoms in the population during the pandemic [1,2,13], particularly among those with pre-existing mental health issues as indicated by this review, underscores the need to dedicate sufficient resources to the mental healthcare system for treatment services. Moreover, clinical surveillance of mental health symptoms during the pandemic and beyond is essential to empowering primary care providers, family, caregivers, and patients themselves to target screening and prevention efforts to those most at risk of a mental health crisis. This can only be achieved through appropriate funding for monitoring and analysis of health system data and mental health research more broadly. During the pandemic, many mental health treatments shifted from in-person patient-facing care to virtual care [86]. Through both self-report and review of health service administrative data, future research should also assess the extent to which patients were able to access needed care and evaluate the effectiveness of its virtual delivery.

Summated characteristics of relevant studies conducted in the first year of the pandemic also have important implications. With data from just 26 countries and five continents worldwide, it is clear that research on symptoms of common mental disorder among those with pre-existing mental health issues lacked geographical representation. Moreover, the bulk of studies were conducted in high-income countries, with little exploration in middle-income countries and no exploration in low-income countries. The review also found that studies reported primarily cross-sectional online surveys, often examining general population samples. More diverse study designs from a broader geographic range, including more low- and middle-income countries, are needed.

There has been an unprecedented proliferation of research related to the COVID-19 pandemic, including studies of its impact on mental health. For this reason, further reviews of this expansive literature will be essential to track and synthesize relevant findings and ensure that they appropriately inform policy and practice. A review of research published in subsequent years of the pandemic is warranted, and may uncover a shift in study design, methodology, study instruments, and sample, as researchers became better positioned to conduct more complex research through government funding for pandemic focused study. This anticipated rise in diversity, complexity, and rigour of pandemic-related studies will augment the potential value of assessing study quality in future reviews, supporting adoption of a *systematic* review approach [84]. Additional reviews should be conducted on research of other mental health diagnoses, to assess if individuals afflicted are also more vulnerable to worsening of related symptoms and/or to depressive, anxiety, and stressor-related symptoms, as assessed here. Finally, a review should be conducted to examine research of the intersection of social determinants of health with pre-existing mental illness as a vulnerability to the mental health impacts of the pandemic. Data have emerged to suggest that marginalized groups, including those who are racialized or economically disadvantaged, have experienced greater mental health effects of the pandemic [87,88]. This was beyond the scope of the current review, but could further refine our ability to target screening, prevention, and treatment within the mental healthcare system.

### Conclusion

The COVID-19 pandemic has had a profound impact on the mental health of the global population. Findings of the current review suggest that individuals with pre-existing mental health issues were at greater risk of adverse mental health impacts of the pandemic. These findings underscore the need for improved support of the mental healthcare system and continued mental health research, including reviews of pandemic effects on individuals with other mental health diagnoses and reviews of research published in subsequent years of the pandemic.

## Supporting information

S1 Checklist PRISMA-ScR (Preferred Reporting Items for Systematic reviews and Meta-Analyses extension for Scoping Reviews) 2018 checklist: Recommended items to address in a scoping review protocol. (DOC)

## References

[1] Ahmed M. Z., Ahmed O., Aibao Z., Hanbin S., Siyu L., & Ahmad A. (2020). Epidemic of COVID-19 in China and associated psychological problems. *Asian Journal of Psychiatry*, 51, 102092. doi: 10.1016/j.ajp.2020.102092 32315963 PMC7194662

[2] Hossain M. M., Tasnim S., Sultana A., Faizah F., Mazumder H., Zou L., et al. (2020). Epidemiology of mental health problems in COVID-19: a review [version 1; peer review: 2 approved]. *F1000Research 2020*, 9, 636. 10.12688/f1000research.24457.1. PMC7549174 33093946

[3] Holmes E. A., O’Connor R. C., Perry V. H., Tracey I., Wessely S., Arseneault L., et al. (2020). Multidisciplinary research priorities for the COVID-19 pandemic: a call for action for mental health science. *The Lancet Psychiatry*, 7(6), 547–560. doi: 10.1016/S2215-0366(20)30168-1 32304649 PMC7159850

[4] Di Nicola M., Dattoli L., Moccia L., Pepe M., Janiri D., Fiorillo A., et al. (2020). Serum 25-hydroxyvitamin D levels and psychological distress symptoms in patients with affective disorders during the COVID-19 pandemic. *Psychoneuroendocrinology*, 122, 104869. doi: 10.1016/j.psyneuen.2020.104869 32956989 PMC7489337

[5] Lahav Y. (2020). Psychological distress related to COVID-19 –The contribution of continuous traumatic stress. *Journal of Affective Disorders*, 277, 129–137. doi: 10.1016/j.jad.2020.07.141 32818776 PMC7416772

[6] Hao F., Tan W., Jiang L., Zhang L., Zhao X., Zou Y., et al. (2020). Do psychiatric patients experience more psychiatric symptoms during COVID-19 pandemic and lockdown? A case-control study with service and research implications for immunopsychiatry. *Brain*, *Behavior*, *and Immunity*, 87, 100–106. doi: 10.1016/j.bbi.2020.04.069 32353518 PMC7184991

[7] Liu C. H., Erdei C., & Mittal L. (2021). Risk factors for depression, anxiety, and PTSD symptoms in perinatal women during the COVID-19 Pandemic. *Psychiatry Research*, 295, 113552. doi: 10.1016/j.psychres.2020.113552 33229122 PMC7904099

[8] Muruganandam P., Neelamegam S., Menon V., Alexander J., & Chaturvedi S. K. (2020). COVID-19 and severe mental illness: Impact on patients and its relation with their awareness about COVID-19. *Psychiatry Research*, 291, 113265. doi: 10.1016/j.psychres.2020.113265 32763536 PMC7322460

[9] American Psychiatric Association. (2013). *Diagnostic and statistical manual of mental disorders* (5th ed.). Arlington, VA : American Psychiatric Association. Dsm.psychiatryonline.org.

[10] World Health Organization. (1993). *The ICD-10 classification of mental and behavioural disorders*. Genève, Switzerland: World Health Organization.

[11] Hawryluck L., Gold W. L., Robinson S., Pogorski S., Galea S., & Styra R. (2004). SARS control and psychological effects of quarantine, Toronto, Canada. *Emerging Infectious Diseases*, 10(7), 1206–1212. doi: 10.3201/eid1007.030703 15324539 PMC3323345

[12] Maunder R. G., Lancee W. J., Balderson K. E., Bennett J. P., Borgundvaag B., Evans S., et al. (2006). Long-term psychological and occupational effects of providing hospital healthcare during SARS outbreak. *Emerging Infectious Diseases*, 12(12), 1924–1932. doi: 10.3201/eid1212.060584 17326946 PMC3291360

[13] Rajkumar R. P. (2020). COVID-19 and mental health: A review of the existing literature. *Asian Journal of Psychiatry*, 52, 102066. doi: 10.1016/j.ajp.2020.102066 32302935 PMC7151415

[14] Peters M. D. J., Godfrey C. M., Khalil H., McInerney P., Parker D., & Soares C. B. (2015). Guidance for conducting systematic scoping reviews. *International Journal of Evidence-Based Healthcare*, 13, 141–146. doi: 10.1097/XEB.0000000000000050 26134548

[15] Arksey H., & O’Malley L. 2005. Scoping studies: towards a methodological framework. *International Journal of Social Research Methodology*, 8(1), 19–32. 10.1080/1364557032000119616.

[16] Tricco A. C., Lillie E., Zarin W., & et al. 2018. PRISMA Extension for Scoping Reviews (PRISMA-ScR): Checklist and explanation. *Annals of Internal Medicine*, 169(7), 467–473. https://www.acpjournals.org/doi/10.7326/M18-0850. 30178033 10.7326/M18-0850

[17] Alonso J., Vilagut G., Mortier P., Ferrer M., Alayo I., Aragón-Peña A., et al. (2021). Mental health impact of the first wave of COVID-19 pandemic on Spanish healthcare workers: A large cross-sectional survey. *Revista de Psiquiatria y Salud Mental*, 14(2). 10.1016/j.rpsm.2020.12.001. PMC10068024 34127211

[18] Aragona M., Tumiati M. C., Ferrari F., Viale S., Nicolella G., Barbato A., et al. (2021). Psychopathological effects of the Coronavirus (Sars-CoV-2) imposed lockdown on vulnerable patients in treatment in a mental health outpatient department for migrants and individuals in poor socioeconomic conditions. *International Journal of Social Psychiatry*, 1–7. 10.1177/0020764020988572. 33438510

[19] Asmundson G. J. G., Paluszek M. M., Landry C. A., Rachor G. S., McKay D., & Taylor S. (2020). Do pre-existing anxiety-related and mood disorders differentially impact COVID-19 stress responses and coping? *Journal of Anxiety Disorders*, 74, 102271. doi: 10.1016/j.janxdis.2020.102271 32673930 PMC7342169

[20] Ballestero M. F. M., Furlanetti L., & de Oliveira R. S. (2020). Pediatric neurosurgery during the COVID-19 pandemic: update and recommendations from the Brazilian Society of Pediatric Neurosurgery. *Neurosurgical Focus*, 49(6), E2. doi: 10.3171/2020.9.FOCUS20703 33260125

[21] Barros M. B. de A, Lima M. G., Malta D. C. Szwarcwald C. L., Azevedo R. C. S. de, Romero D., et al. (2020). Report on sadness/depression, nervousness/anxiety and sleep problems in the Brazilian adult population during the COVID-19 pandemic. *Epidemiologia E Serviços de Saúde*, 29(4), e2020427. 10.1590/s1679-49742020000400018. 32844918

[22] Bruffaerts R., Voorspoels W., Jansen L., Kessler R. C., Mortier P., Vilagut G., et al. (2021). Suicidality among healthcare professionals during the first COVID19 wave. *Journal of Affective Disorders*, 283, 66–70. doi: 10.1016/j.jad.2021.01.013 33524660 PMC7832920

[23] Bruine de Bruin W. (2020). Age differences in COVID-19 risk perceptions and mental health: Evidence from a national U.S. survey conducted in March 2020. *The Journals of Gerontology*: *Series B*, 76(2), e24–e29. 10.1093/geronb/gbaa074. PMC7542924 32470120

[24] Chaix B., Delamon G., Guillemassé A., Brouard B., & Bibault J.-E. (2020). Psychological distress during the COVID-19 pandemic in France: a national assessment of at-risk populations. *General Psychiatry*, 33(6), e100349. doi: 10.1136/gpsych-2020-100349 34192239 PMC7692000

[25] Cheema M., Mitrev N., Hall L., Tiongson M., Ahlenstiel G., & Kariyawasam V. (2021). Depression, anxiety and stress among patients with inflammatory bowel disease during the COVID-19 pandemic: Australian national survey. *BMJ Open Gastroenterology*, 8(1), e000581. doi: 10.1136/bmjgast-2020-000581 33579729 PMC7883604

[26] Conte G., Baglioni V., Valente F., Chiarotti F., & Cardona F. (2020). Adverse mental health impact of the COVID-19 lockdown in Individuals with Tourette syndrome in Italy: An online survey. *Frontiers in Psychiatry*, 11, 583744. doi: 10.3389/fpsyt.2020.583744 33329125 PMC7734024

[27] Costa M., Pavlo A., Reis G., Ponte K., & Davidson L. (2020). COVID-19 concerns among persons with mental illness. *Psychiatric Services*, 71(11), 1188–1190. doi: 10.1176/appi.ps.202000245 32878542

[28] De Pietri S., & Chiorri C. (2021). Early impact of COVID-19 quarantine on the perceived change of anxiety symptoms in a non-clinical, non-infected Italian sample. *Journal of Affective Disorders Reports*, 4, 100078. 10.1016/j.jadr.2021.100078. 33469571 PMC7808247

[29] Duarte H., Daros Vieira R., Cardozo Rocon P., Andrade A. C. D. S., Wittmer V. L., Capellini V. K., et al. (2021). Factors associated with Brazilian physical therapists’ perception of stress during the COVID-19 pandemic: a cross-sectional survey. *Psychology*, *Health & Medicine*, 1–12. doi: 10.1080/13548506.2021.1875133 33487038

[30] Einvik G., Dammen T., Ghanima W., Heir T., & Stavem K. (2021). Prevalence and risk factors for post-traumatic stress in hospitalized and non-hospitalized COVID-19 patients. *International Journal of Environmental Research and Public Health*, 18(4), 1–12. doi: 10.3390/ijerph18042079 33672759 PMC7924607

[31] Fallon V., Davies S. M., Silverio S. A., Jackson L., De Pascalis L., & Harrold J. A. (2021). Psychosocial experiences of postnatal women during the COVID-19 pandemic. A UK-wide study of prevalence rates and risk factors for clinically relevant depression and anxiety. *Journal of Psychiatric Research*, 136, 157–166. doi: 10.1016/j.jpsychires.2021.01.048 33596462 PMC8635302

[32] Fountoulakis K. N., Apostolidou M. K., Atsiova M. B., Filippidou A. K., Florou A. K., Gousiou D. S., et al. (2021). Self-reported changes in anxiety, depression and suicidality during the COVID-19 lockdown in Greece. *Journal of Affective Disorders*, 279, 624–629. doi: 10.1016/j.jad.2020.10.061 33190113 PMC7605790

[33] Fu R., & Zhang Y. (2020). Case report of a patient with suspected COVID-19 with depression and fever in an epidemic stress environment. *General Psychiatry*, 33(3), e100218. doi: 10.1136/gpsych-2020-100218 34192229 PMC7276235

[34] Gao Y., Sun F., Jiang W., Fang Y., Yue L., Lin X., et al. (2020). Beliefs towards the COVID-19 pandemic among patients with emotional disorders in China. *General Psychiatry*, 33(3), e100231. doi: 10.1136/gpsych-2020-100231 32574346 PMC7287490

[35] Ge F., Zhang D., Wu L., & Mu H. (2020). Predicting psychological state among Chinese undergraduate students in the COVID-19 epidemic: A longitudinal study using a machine learning. *Neuropsychiatric Disease and Treatment*, 16, 2111–2118. doi: 10.2147/NDT.S262004 32982249 PMC7505704

[36] Gobbi S., Płomecka M. B., Ashraf Z., Radziński P., Neckels R., Lazzeri S., et al. (2020). Worsening of preexisting psychiatric conditions during the COVID-19 pandemic. *Frontiers in Psychiatry*, 11, 581426. doi: 10.3389/fpsyt.2020.581426 33391049 PMC7772353

[37] González-Blanco L., Santo F., García-Álvarez L., De La Fuente-Tomás L., Lacasa C., Paniagua G., et al. (2020). COVID-19 lockdown in people with severe mental disorders in Spain: Do they have a specific psychological reaction compared with other mental disorders and healthy controls? *Schizophrenia Research*, 223, 192–198. doi: 10.1016/j.schres.2020.07.018 32771308 PMC7381938

[38] Hamam A. A., Milo S., Mor I., Shaked E., Eliav A. S., & Lahav Y. (2021). Peritraumatic reactions during the COVID-19 pandemic–The contribution of posttraumatic growth attributed to prior trauma. *Journal of Psychiatric Research*, 132, 23–31. doi: 10.1016/j.jpsychires.2020.09.029 33038562 PMC7525333

[39] Hamm M. E., Brown P. J., Karp J. F., Lenard E., Cameron F., Dawdani A., et al. (2020). Experiences of American older adults with pre-existing depression during the beginnings of the COVID-19 pandemic: A multicity, mixed-methods study. *The American Journal of Geriatric Psychiatry*, 28(9), 924–932. doi: 10.1016/j.jagp.2020.06.013 32682619 PMC7305766

[40] Hao F., Tam W., Hu X., Tan W., Jiang L., Jiang X., et al. (2020). A quantitative and qualitative study on the neuropsychiatric sequelae of acutely ill COVID-19 inpatients in isolation facilities. *Translational Psychiatry*, 10(1), 1–14. 10.1038/s41398-020-01039-2. 33077738 PMC7570419

[41] Hodžić N., Hasanović M., & Pajević I. (2020). COVID-19 affected mental health of at-riks groups of psychiatric patients: Two case reports. *Psychiatria Danubina*, 32(2), 294–299. 10.24869/psyd.2020.294. 32796801

[42] Holingue C., Kalb L., Riehm K., Bennett D., Kapteyn A., Veldhuis C., et al. (2020). Mental distress in the United States at the beginning of the COVID-19 pandemic. *American Journal of Public Health*, 110(11). 10.2105/AJPH.2020.305857. PMC7542294 32941066

[43] Hölzle P., Aly L., Frank W., Förstl H., & Frank A. (2020). COVID-19 distresses the depressed while schizophrenic patients are unimpressed: A study on psychiatric inpatients. *Psychiatry Research*, 291, 113175. doi: 10.1016/j.psychres.2020.113175 32535514 PMC7274101

[44] Janiri D., Petracca M., Moccia L., Tricoli L., Piano C., Bove F., et al. (2020). COVID-19 pandemic and psychiatric symptoms: The impact on Parkinson’s Disease in the elderly. *Frontiers in Psychiatry*, 11, 581144. doi: 10.3389/fpsyt.2020.581144 33329124 PMC7728715

[45] Jefsen O. H., Rohde C., Nørremark B., & Østergaard S. D. (2020). COVID-19-related self-harm and suicidality among individuals with mental disorders. *Acta Psychiatrica Scandinavica*, 142(2), 152–153. doi: 10.1111/acps.13214 32659855 PMC7404949

[46] Jefsen O. H., Rohde C., Nørremark B., & Østergaard S. D. (2020). Editorial Perspective: COVID‐19 pandemic‐related psychopathology in children and adolescents with mental illness. *Journal of Child Psychology and Psychiatry*, 62(6), 798–800. doi: 10.1111/jcpp.13292 32779748 PMC7361472

[47] Karaahmet E., Angın Ü., Yılmaz O., Deniz D., & Konuk N. (2021). Assessment of psychometric characteristics of the Coronavirus Anxiety Scale in patients with preexisting psychiatric disorders. *Death Studies*, 1–5. doi: 10.1080/07481187.2021.1876184 33494656

[48] Khosravani V., Asmundson G. J. G., Taylor S., Sharifi Bastan F., & Samimi Ardestani S. M. (2021). The Persian COVID stress scales (Persian-CSS) and COVID-19-related stress reactions in patients with obsessive-compulsive and anxiety disorders. *Journal of Obsessive-Compulsive and Related Disorders*, 28, 100615. doi: 10.1016/j.jocrd.2020.100615 33354499 PMC7746142

[49] Kwong A. S. F., Pearson R. M., Adams M. J., Northstone K., Tilling K., Smith D., et al. (2020). Mental health before and during the COVID-19 pandemic in two longitudinal UK population cohorts. *The British Journal of Psychiatry*, 218(6), 1–10. 10.1192/bjp.2020.242. PMC7844173 33228822

[50] Li W., Zhao N., Yan X., Zou S., Wang H., Li Y., et al. (2021). The prevalence of depressive and anxiety symptoms and their associations with quality of life among clinically stable older patients with psychiatric disorders during the COVID-19 pandemic. *Translational Psychiatry*, 11, 75. doi: 10.1038/s41398-021-01196-y 33500389 PMC7835649

[51] Liao Y. H., Fan B. F., Zhang H. M., Guo L., Lee Y., Wang W. X., et al. (2021). The impact of COVID-19 on subthreshold depressive symptoms: a longitudinal study. *Epidemiology and Psychiatric Sciences*, 30, e20. doi: 10.1017/S2045796021000044 33583474 PMC7985630

[52] Mehra A., Rani S., Sahoo S., Parveen S., Singh A. P., Chakrabarti S., et al. (2020). A crisis for elderly with mental disorders: Relapse of symptoms due to heightened anxiety due to COVID-19. *Asian Journal of Psychiatry*, 51, 102114. doi: 10.1016/j.ajp.2020.102114 32334406 PMC7166027

[53] Mink van der Molen D. R, Bargon C. A., Batenburg M. C. T, Gal R., Young-Afat D. A., van Stam, L. E., et al. (2021). (Ex-)breast cancer patients with (pre-existing) symptoms of anxiety and/or depression experience higher barriers to contact health care providers during the COVID-19 pandemic. *Breast Cancer Research and Treatment*, 186(2), 577–583. doi: 10.1007/s10549-021-06112-y 33598879 PMC7889408

[54] Mortier P., Vilagut G., Ferrer M., Serra C., Dios Molina J., López‐Fresneña N., et al. (2021). Thirty‐day suicidal thoughts and behaviors among hospital workers during the first wave of the Spain COVID‐19 outbreak. *Depression and Anxiety*, 38(5), 528–544. doi: 10.1002/da.23129 33393724 PMC8246904

[55] Mortier P., Vilagut G., Ferrer M., Alayo I., Bruffaerts R., Cristóbal-Narváez P., et al. (2021). Thirty-day suicidal thoughts and behaviours in the Spanish adult general population during the first wave of the Spain COVID-19 pandemic. *Epidemiology and Psychiatric Sciences*, 30, e19. doi: 10.1017/S2045796021000093 34187614 PMC7925988

[56] Padovan-Neto F. E., Lee S. A., Guimarães R. P., Godoy L. D., Costa H. B., Zerbini F. L. S., et al. (2023). Brazilian adaptation of the Coronavirus Anxiety Scale: A psychometric investigation of a measure of coronaphobia. *Omega (Westport)*, 86(3), 769–787. doi: 10.1177/0030222821991325 33530891 PMC9810823

[57] Pan K.-Y., Kok A. A. L., Eikelenboom M., Horsfall M., Jörg F., Luteijn R. A., et al. (2021). The mental health impact of the COVID-19 pandemic on people with and without depressive, anxiety, or obsessive-compulsive disorders: a longitudinal study of three Dutch case-control cohorts. *The Lancet Psychiatry*, 8(2), 121–129. doi: 10.1016/S2215-0366(20)30491-0 33306975 PMC7831806

[58] Pizzirusso M., Carrion-Park C., Clark U. S., Gonzalez J., Byrd D., & Morgello S. (2021). Physical and mental health screening in a New York City HIV cohort during the COVID-19 pandemic: A preliminary report. *Journal of Acquired Immune Deficiency Syndromes*, 86(3), e54–e60. doi: 10.1097/QAI.0000000000002564 33148994 PMC7878300

[59] Plunkett R., Costello S., McGovern M., McDonald C., & Hallahan B. (2020). Impact of the COVID-19 pandemic on patients with pre-existing anxiety disorders attending secondary care. *Irish Journal of Psychological Medicine*, 38(2), 123–131. doi: 10.1017/ipm.2020.75 32507119 PMC7324660

[60] Prazeres F., Passos L., Simões J. A., Simões P., Martins C., & Teixeira A. (2020). COVID-19-related fear and anxiety: Spiritual-religious coping in healthcare workers in Portugal. *International Journal of Environmental Research and Public Health*, 18(1), 220. doi: 10.3390/ijerph18010220 33396750 PMC7794895

[61] Quittkat H. L., Düsing R., Holtmann F.-J., Buhlmann U., Svaldi J., & Vocks S. (2020). Perceived impact of Covid-19 across different mental disorders: A study on disorder-specific symptoms, psychosocial stress and behavior. *Frontiers in Psychology*, 11, 586246. doi: 10.3389/fpsyg.2020.586246 33281685 PMC7705217

[62] Ravaldi C., Ricca V., Wilson A., Homer C., & Vannacci A. (2020). Previous psychopathology predicted severe COVID-19 concern, anxiety, and PTSD symptoms in pregnant women during “lockdown” in Italy. *Archives of Women’s Mental Health*, 23(6), 783–786. doi: 10.1007/s00737-020-01086-0 33215247 PMC7677012

[63] Rogers A. A., Ha T., & Ockey S. (2021). Adolescents’ perceived socio-emotional impact of COVID-19 and implications for mental health: Results from a U.S.-based mixed-methods study. *Journal of Adolescent Health*, 68(1), 43–52. doi: 10.1016/j.jadohealth.2020.09.039 33143986 PMC7605752

[64] Rutherford B. R., Choi C. J., Chrisanthopolous M., Salzman C., Zhu C., Montes-Garcia C., et al. (2021). The COVID-19 pandemic as a traumatic stressor: Mental health responses of older adults with chronic PTSD. *The American Journal of Geriatric Psychiatry*, 29(2), 105–114. doi: 10.1016/j.jagp.2020.10.010 33153871 PMC7582036

[65] Saraswathi I., Saikarthik J., Senthil Kumar K., Madhan Srinivasan K., Ardhanaari M., & Gunapriya R. (2020). Impact of COVID-19 outbreak on the mental health status of undergraduate medical students in a COVID-19 treating medical college: a prospective longitudinal study. *PeerJ*, 8, e10164. doi: 10.7717/peerj.10164 33088628 PMC7571415

[66] Serafini R. A., Powell S. K., Frere J. J., Saali A., Krystal H. L., Kumar V., et al. (2021). Psychological distress in the face of a pandemic: An observational study characterizing the impact of COVID-19 on immigrant outpatient mental health. *Psychiatry Research*, 295, 113595. doi: 10.1016/j.psychres.2020.113595 33296817 PMC7805919

[67] Sharma A. J., & Subramanyam M. A. (2020). A cross-sectional study of psychological wellbeing of Indian adults during the Covid-19 lockdown: Different strokes for different folks. *PLOS One*, 15(9), e0238761. doi: 10.1371/journal.pone.0238761 32881946 PMC7470332

[68] Sherman A. C., Williams M. L., Amick B. C., Hudson T. J., & Messias E. L. (2020). Mental health outcomes associated with the COVID-19 pandemic: Prevalence and risk factors in a southern US state. *Psychiatry Research*, 293, 113476. doi: 10.1016/j.psychres.2020.113476 33198047 PMC7513769

[69] Solé B., Verdolini N., Amoretti S., Montejo L., Rosa A. R., Hogg B., et al. (2021). Effects of the COVID-19 pandemic and lockdown in Spain: comparison between community controls and patients with a psychiatric disorder. Preliminary results from the BRIS-MHC STUDY. *Journal of Affective Disorders*, 281, 13–23. doi: 10.1016/j.jad.2020.11.099 33279864 PMC7683299

[70] Solomou I., & Constantinidou F. (2020). Prevalence and predictors of anxiety and depression symptoms during the COVID-19 pandemic and compliance with precautionary measures: Age and sex matter. *International Journal of Environmental Research and Public Health*, 17(14), 4924. doi: 10.3390/ijerph17144924 32650522 PMC7400373

[71] Sorokin M. Y., Kasyanov E. D., Rukavishnikov G. V., Makarevich O. V., Neznanov N. G., Lutova N. B., et al. (2020). Structure of anxiety associated with СOVID-19 pandemic: the online survey results. *Bulletin of Russian State Medical University*, 70–76. 10.24075/vrgmu.2020.030.

[72] Sun Q., Qin Q., Basta M., Chen B., & Li Y. (2021). Psychological reactions and insomnia in adults with mental health disorders during the COVID-19 outbreak. *BMC Psychiatry*, 21, 19. doi: 10.1186/s12888-020-03036-7 33419411 PMC7791151

[73] Thomb B. D., Kwak kenbos L, Henry R. S, Carrier M.-E., Patten S, Harb S., et al. (2020). Changes in mental health symptoms from pre-COVID-19 to COVID-19 among participants with systemic sclerosis from four countries: A Scleroderma Patient-centered Intervention Network (SPIN) Cohort study. *Journal of Psychosomatic Research*, 139, 110262. doi: 10.1016/j.jpsychores.2020.110262 33070043 PMC7532799

[74] Torales J., Ríos-González C., Barrios I., O’Higgins M., González I., García O., et al. (2020). Self-perceived stress during the quarantine of COVID-19 pandemic in Paraguay: An exploratory survey. *Frontiers in Psychiatry*, 11, 558691. doi: 10.3389/fpsyt.2020.558691 33192674 PMC7649175

[75] Vissink C. E., van Hell H. H., Galenkamp N., & van Rossum I. W. (2021). The effects of the COVID-19 outbreak and measures in patients with a pre-existing psychiatric diagnosis: A cross-sectional study. *Journal of Affective Disorders Reports*, 4, 100102. doi: 10.1016/j.jadr.2021.100102 33558866 PMC7848531

[76] Werneck A. O., Silva D. R., Malta D. C., Souza-Júnior P. R. B., Azevedo L. O., Barros M. B. A., et al. (2021). Physical inactivity and elevated TV-viewing reported changes during the COVID-19 pandemic are associated with mental health: A survey with 43,995 Brazilian adults. *Journal of Psychosomatic Research*, 140, 110292. doi: 10.1016/j.jpsychores.2020.110292 33227555 PMC7654295

[77] Woon L. S.-C., Sidi H., Nik Jaafar N. R., & Leong Bin Abdullah M. F. I. (2020). Mental health status of university healthcare workers during the COVID-19 pandemic: A post–movement lockdown assessment. *International Journal of Environmental Research and Public Health*, 17(24), 9155. doi: 10.3390/ijerph17249155 33302410 PMC7762588

[78] Zhu J., Sun L., Zhang L., Wang H., Fan A., Yang B., et al. (2020). Prevalence and influencing factors of anxiety and depression symptoms in the first-line medical staff fighting against COVID-19 in Gansu. *Frontiers in Psychiatry*, 11, 386. doi: 10.3389/fpsyt.2020.00386 32411034 PMC7202136

[79] World Health Organization. (2020). *Coronavirus disease 2019 (COVID-19) Situation Report– 86*. https://www.who.int/docs/default-source/coronaviruse/situation-reports/20200415-sitrep-86-covid-19.pdf.

[80] Murphy L., Markey K., O’Donnell C., Moloney M., & Doody O. (2021). The impact of the COVID-19 pandemic and its related restrictions on people with pre-existent mental health conditions: A scoping review. *Archives of Psychiatric Nursing*, 35, 375–394. doi: 10.1016/j.apnu.2021.05.002 34176579 PMC9759111

[81] Neelam K., Duddu V., Anyim N., Neelam J., & Lewis S. (2021). Pandemics and pre-existing mental illness: A systematic review and meta-analysis. *Brain*, *Behavior*, *& Immunity–Health*, 10, 100177. doi: 10.1016/j.bbih.2020.100177 33251527 PMC7683956

[82] Vindegaard N., & Eriksen Benros M. (2020). COVID-19 pandemic and mental health consequences: Systematic review of the current evidence. *Brain*, *Behavior*, *and Immunity*, 89, 531–542. doi: 10.1016/j.bbi.2020.05.048 32485289 PMC7260522

[83] Patten S. B., Kutcher S., Streiner D., Gratzer D., Kurdyak P., & Yatham L. (2021). Population mental health and COVID-19: Why do we know so little? *The Canadian Journal of Psychiatry*, 66(9), 782–784. doi: 10.1177/07067437211010523 33871302 PMC8495301

[84] Munn Z., Peters M. D. J., Stern C., Tufanaru C., McArthur A., & Aromataris E. (2018). Systematic review or scoping review? Guidance for authors when choosing between a systematic or scoping review approach. *BMC Medical Research Methodology*, 18, 143. doi: 10.1186/s12874-018-0611-x 30453902 PMC6245623

[85] Pham M. T., Rajić A., Greig J. D., Sargeant J. M., Papadopoulos A., & McEwan S. A. (2014). A scoping review of scoping reviews: advancing the approach and enhancing the consistency. *Research Synthesis Methods*, 5, 371–385. doi: 10.1002/jrsm.1123 26052958 PMC4491356

[86] Asmundson G. J. G., Blackstock C., Bourque M. C., Brimacombe G., Crawford A., Deacon S. H., et al. (2020). Easing the disruption of COVID-19: supporting the mental health of the people of Canada-October 2020-an RSC policy briefing. *FACETS*, 5, 1071–1098. 10.1139/facets-2020-0082.

[87] McNeely C. L., Schintler L. A., & Stabile B. (2020). Social determinants and COVID-19 disparities: Differential pandemic effects and dynamics. *World Medical and Health Policy*, 12(3), 206–217.

[88] Raina P., Wolfson C., Griffith L., Kirkland S., McMillan J., Basta N., et al. (2021). A longitudinal analysis of the impact of the COVID-19 pandemic on the mental health of middle-aged and older adults from the Canadian Longitudinal Study on Aging. *Nature Aging*, 1, 1137–1147. doi: 10.1038/s43587-021-00128-1 37117519

